# Circulating RNA biomarkers in diffuse large B-cell lymphoma: a systematic review

**DOI:** 10.1186/s40164-021-00208-3

**Published:** 2021-02-16

**Authors:** Philippe Decruyenaere, Fritz Offner, Jo Vandesompele

**Affiliations:** 1 Department of Hematology, Ghent University Hospital, 9K12, Campus UZ Ghent, Corneel Heymanslaan 10, 9000 Ghent, Belgium; 2 OncoRNALab, Cancer Research Institute Ghent (CRIG), Ghent, Belgium; 3 Department of Biomolecular Medicine, Ghent University, Ghent, Belgium

**Keywords:** DLBCL, Diffuse large B-cell lymphoma, Biomarkers, Liquid biopsy, Extracellular RNA

## Abstract

Diffuse large B-cell lymphoma (DLBCL) is the most common histological subtype of non-Hodgkin’s lymphomas (NHL). DLBCL is an aggressive malignancy that displays a great heterogeneity in terms of morphology, genetics and biological behavior. While a sustained complete remission is obtained in the majority of patients with standard immunochemotherapy, patients with refractory of relapsed disease after first-line treatment have a poor prognosis. This patient group represents an important unmet need in lymphoma treatment. In recent years, improved understanding of the underlying molecular pathogenesis had led to new classification and prognostication tools, including the development of cell-free biomarkers in liquid biopsies. Although the majority of studies have focused on the use of cell-free fragments of DNA (cfDNA), there has been an increased interest in circulating-free coding and non-coding RNA, including messenger RNA (mRNA), microRNA (miRNA), long non-coding RNA (lncRNA) and circular RNA (circRNA), as well as RNA encapsulated in extracellular vesicles or tumor-educated platelets (TEPs). We performed a systematic search in PubMed to identify articles that evaluated circulating RNA as diagnostic, subtype, treatment response or prognostic biomarkers in a human DLBCL population. A total of 35 articles met the inclusion criteria. The aim of this systematic review is to present the current understanding of circulating RNA molecules as biomarker in DLBCL and to discuss their future potential.

## Introduction

A lymphoma is a hematopoietic malignancy that develops in the lymphoid tissue. The diffuse large-cell B-cell lymphoma (DLBCL) is the most common histological subtype of non-Hodgkin’s lymphomas (NHL), representing approximately 25% of new diagnoses. It can occur de novo or as a result of transformation from different types of low-grade B-cell lymphomas such as chronic lymphatic leukemia (CLL), lymphoplasmocytic lymphoma, follicular lymphoma and (splenic) marginal zone lymphoma. With the standard treatment of R-CHOP immunochemotherapy (rituximab, cyclophosphamide, vincristine, doxorubicin and prednisone), sustained remission can be obtained in approximately 60–70% of patients [1]. However, patients with refractory or relapsed disease after first-line treatment have a reserved prognosis with a 5-year survival rate of only 20%, despite second-line treatments [2, 3]. This subgroup represents an important unmet need in lymphoma treatment. The prognostic differences in terms of response and survival reflect the heterogeneity of different subgroups of DLBCL with respect to morphology, genetics and biological behavior.

A liquid biopsy is the process of investigating tumor-derived cells or biomaterials like cell-free nucleic acids, metabolites, proteins or extracellular vesicles through biofluid sampling such as peripheral blood, urine, saliva, and cerebral spinal fluid, without the need of a tissue biopsy. In recent years, there has been a major interest and advance in the use of liquid biopsy in lymphoma management due to its non-invasive nature, its ability to reflect spatial inter- and intra-tumor heterogeneity, and the possibility of repeated measurements through longitudinal profiling [4].

The vast majority of studies have focused on the use of circulating cell-free DNA fragments (cfDNA), a proportion of which is derived from lymphoma cells (circulating tumor DNA; ctDNA). Studies have shown that cfDNA plasma concentration is associated with lymphoma aggressiveness, tumor volume and disease stage, may predict therapy response, and has prognostic value in assessing progressive, relapsing or minimal residual disease. Moreover, cfDNA is able to represent the clonality and mutational burden of DLBCL and can discriminate between different cell-of-origin (COO) subgroups: germinal center B-cell-like (GCB) and activated B-cell-like (ABC) [4–6]. The COO classification is originally based on gene expression profiling and is routinely implemented through the use of surrogate immunohistochemical techniques, such as the Hans algorithm [7, 8]. Scherer et al. showed a 80% concordance rate with the Hans algorithm using the identification of mutations obtained through liquid biopsies [5]. Several studies have formulated other DLBCL classifications with prognostic implications, based on different mutations and structural variations found in tissue biopsies, one of which could already be reproduced using ctDNA with high concordance [9–15]. These classifications may complement conventional prognostic scores, such as the international prognostic index (IPI), in identifying high-risk subsets of patients [16]. The importance of understanding the heterogeneity in DLBCL was illustrated by several studies that identified subgroups of high-risk patients that could benefit from associating targeted therapy to standard treatment, emphasizing the significance of a personalized medicine approach [17–19].

**Fig. 1:**
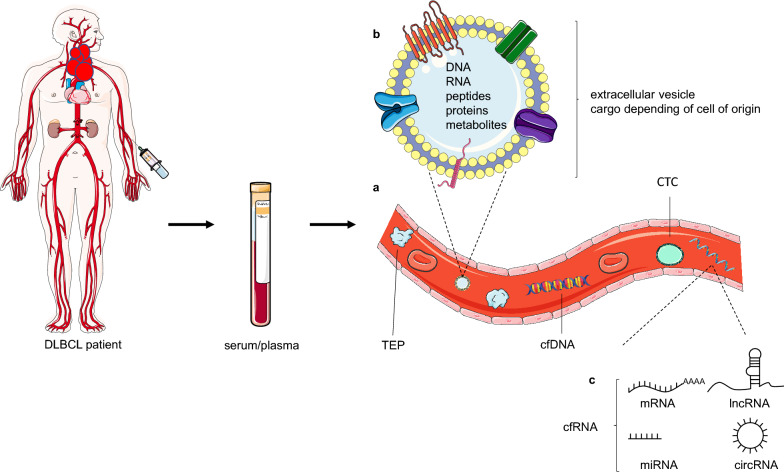
Circulating biomarkers in DBLCL. A schematic representation of circulating biomarkers obtained through blood draw, such as circulating tumor cells (CTC), tumor-educated platelets (TEPs), cell-free DNA (cfDNA) and cell-free RNA (cfRNA).** a** Extracellular vesicles include exosomes, microvesicles and apoptotic bodies. Their cargo reflects the cell of origin and contains proteins, peptides, lipids, cfDNA and cfRNA. **b** CfRNA comprises both coding and non-coding types, including mRNA, miRNA, lncRNA and circRNA.** c** Figure produced using Servier Medical Art

In recent years, there has been an increased interest in other circulating biomarkers, such as circulating tumor cells (CTC) and different forms of circulating-free and extracellular vesicle/platelet-encapsulated coding and non-coding RNA, including messenger RNA (mRNA), microRNA (miRNA), long non-coding RNA (lncRNA), and circular RNA (circRNA) (Fig. 1). Although CTC may provide tumor-specific genomic, transcriptomic, and proteomic information, their analysis is less attractive since DLBCL do not typically present with circulating lymphoma cells (in contrast to mantle cell lymphoma, follicular lymphoma (FL), marginal zone lymphoma, small lymphocytic lymphoma, and a subset of Burkitt lymphoma) [20]. Also, CTC analysis requires a large volume of fresh blood, and is laborious and expensive. The use of cell-free RNA (cfRNA), however, has shown promise as a precision medicine biomarker. Here, we provide a systematic overview of cell-free RNA biomarkers in DLBCL and their future potential in the diagnosis, classification, real-time measurement of response to therapy, and prognosis.

## Materials and methods

**Fig. 2:**
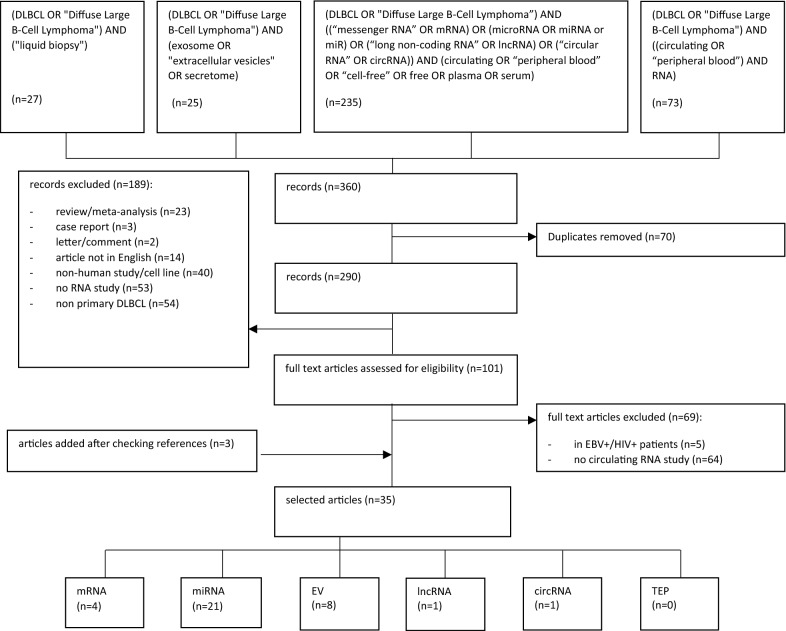
Flowchart of study selection

A systematic search in PubMed was performed to identify articles published between January 01, 1970 and October 31, 2020 using the following strategy (Fig. 2): [(DLBCL OR “Diffuse Large B-Cell Lymphoma”) AND (“liquid biopsy”) OR (DLBCL OR “Diffuse Large B-Cell Lymphoma”) AND (exosome OR “extracellular vesicles” OR secretome) OR (DLBCL OR “Diffuse Large B-Cell Lymphoma”) AND ((“messenger RNA” OR mRNA) OR (microRNA OR miRNA or miR) OR (“long non-coding RNA” OR lncRNA) OR (“circular RNA” OR circRNA)) AND (circulating OR “peripheral blood” OR “cell-free” OR free OR plasma OR serum) OR (DLBCL OR “Diffuse Large B-Cell Lymphoma”) AND ((circulating OR “peripheral blood”) AND RNA)]. Articles were included if they presented independent original studies in a human adult DLBCL population. Reviews and meta-analyses, case reports, letters, comments, and articles not published in English were excluded. We also excluded studies on animals, DLBCL cell lines/xenografts, as well as studies that did not analyze RNA or did not focus on primary DLBCL. After full text assessment, articles investigating patients with additional pathologies or not analyzing circulating RNAs as biomarker in diagnosis, subtype classification, treatment response, or prognosis, were excluded. All references within the selected studies were reviewed in order to identify additional matches.

Each eligible manuscript was assessed independently by two researchers. Disagreements were resolved by consensus. Data extracted from each study included: publication year, technical methodology and type of blood-based fluid analyzed, method of EV purification, number of DLBCL cases and controls, the investigated RNAs as potential biomarkers, and their association with an outcome. In order to define associations between the abundance of the RNAs and the phenotypes, a p-value < 0.05 was considered statistically significant.

## Results

A total of 360 records were initially identified and 290 remained after removing duplicates. Among these, 189 were excluded after abstract revision because they did not meet the inclusion criteria. Based on the full text of 101 articles, 69 articles were excluded because they focused on tissue-derived RNA or on specific patient groups with additional pathologies. After reviewing the references of the identified articles, three additional studies were included. Finally, a total of 35 articles were included and classified into the following groups, depending on the type of circulating RNA studied: mRNA, miRNA, EV, lncRNA, circRNA, and tumor-educated platelets (TEPs) (Fig. 2).

### Cell‐free RNA

Although 80% of the human genome is transcribed into RNA, it has been estimated that protein-coding mRNA only accounts for 1.5% of this output with the remainder being termed non-coding RNA (ncRNA) [21]. As the functions of most ncRNA remain largely unknown, current classifications are based primarily on size and differentiate between short ncRNAs (less than 200 nucleotides, including miRNAs) and large ncRNAs (larger than 200 bases, generally termed lncRNAs, but also including circRNAs) [22].

Circulating-free RNAs are unstable molecules that are quickly degraded by ribonucleases. This was demonstrated in a lability experiment, in which 99% of added naked RNA was degraded after a 15 s incubation time [23]. It is clear that endogenous circulating RNAs are protected by several mechanisms, such as encapsulation within extracellular vesicles (EVs) or they form ribonucleoprotein complexes with RNA-binding proteins such as nucleophosmin, high-density lipoprotein or Argonaute 1 and 2 that protect them from nuclease activity. Although the source and function of circulating RNAs are largely unclarified, RNAs seem to be selectively packaged according to the viability and origin of the cells. While living cells actively release RNA encapsulated in large lipoprotein complexes, such as exosomes or microvesicles (MVs), RNA from dead or dying cells found in blood is associated with apoptotic bodies (ABs) or protein complexes [24–26]. Lastly, circulating RNA can be encapsulated by platelets that, when exposed to tumor cells, can be ‘educated’ by changing their RNA profile, mainly by altering splicing patterns and to lesser extent by ingestion of tumor-derived circulating RNA and EVs. These platelets are referred to as tumor-educated platelets (TEPs) [27–31].

Higher circulating RNA levels have been found in patients with solid and hematological malignancies, compared to patients without tumor. It is believed that cancer cells communicate with surrounding stromal and immune cells via extracellular RNAs, which may contribute to increased proliferation, malignant transformation of surrounding cells, angiogenesis, escape from the immune response, and priming of future metastatic niches [25, 26, 32]. Although being technically more challenging than cfDNA, the analysis of cfRNA may therefore have certain advantages as biomarker. Since these molecules mediate or influence intercellular communication, they may lead to an improved understanding of key pathways involved in normal differentiation, as well as in lymphoma initiation and transformation. Moreover, differences in cfRNA patterns may reflect functional, longitudinal changes in both the tumor and the non-malignant compartment during disease course or treatment. This specific and dynamic characterization, which incorporates the crosstalk between tumor and environment, may guide the development of individualized diagnostic and therapeutic options, especially in genetically heterogeneous diseases such as DLBCL.

Up to this date, we found 35 studies that have examined the serum/plasma levels of RNA and its association with clinical and pathological characteristics of DLBCL, suggesting that cell-free RNA may provide biomarkers for the diagnosis, classification, measurement of response to therapy, and prognosis.

### Messenger RNA

Messenger RNA (mRNA) is the result of DNA transcription and possesses a critical intermediary role in intracellular protein translation, reflecting both the genomic and the homeostatic state of the cell. These properties make mRNA a potentially interesting target for liquid biopsy. In 1991, Smith et al. used PCR analysis on tyrosinase mRNA, a tissue-specific gene in melanocytes, to detect the presence of circulating melanoma cells [33]. This was followed by the identification of tyrosinase mRNA in the serum of melanoma patients [34]. Over the years, circulating tumor mRNAs haven been described in many different tumors and their levels were associated with cancer aggressiveness, disease prognosis and response to chemoradiotherapy [35–39]. Moreover, they have shown potential to guide individualized therapy choice, as was illustrated by the detection of different ALK fusion mRNAs with high sensitivity and specificity in patients with non-small cell lung cancer [40].

Four studies have investigated cell-free mRNAs in DLBCL (Table 1). Garcia et al. showed that G1/S-specific cyclin-D2 (CCND2), MYC proto-oncogene protein (MYC), B-cell lymphoma 2 (BCL2), and LIM domain only 2 (LMO2) mRNAs were significantly higher in DLBCL plasma samples. The presence of circulating MYC or CCND2 mRNA was associated with worse overall survival (OS), especially in low-risk IPI group, and the presence of CCND2, BCL2 or MYC mRNA in patients with complete response (CR) was associated with worse progression-free survival (PFS) [41]. These genes are known to play important roles in lymphomagenesis. CCND2 is a key player in cell division and proliferation, regulated by the phosphatidylinositol 3/kinase-protein kinase B/mammalian target of rapamycin (PI3K/AKT/mTOR) pathway. The MYC gene encodes for a transcription factor that controls numerous biological functions, including proliferation, cell growth, telomerase activity, energy metabolism, differentiation, and apoptosis, as well as DNA replication. BCL2 is a key anti-apoptotic molecule expressed in most tissues but absent in the germinal center, and LMO2 is expressed in all tissues with the exception of mature T-cells and is implicated in angiogenesis, hematopoiesis, and hematopoietic stem cell maintenance [42, 43]. Zhao et al. demonstrated a higher abundance of CREBBP mRNA in DLBCL blood samples as compared with controls. However, no differences were found in OS and PFS. CREBBP is one of the most frequently mutated genes in DLBCL and acts as a tumor suppressor of germinal center-derived lymphomagenesis by promoting transcription, counteracting the inhibition of B-cell lymphoma 6 (BCL6) [44]. Attia et al. showed that a higher abundance of suppressor of cytokine signaling 3 (SOCS-3) mRNA in plasma samples of NHL patients correlates with advanced disease and poor response to treatment. However, no separate analysis of the DLBCL patient subgroup has been performed, limiting its interpretation. SOCS3, among other functions, inhibits the Janus kinase (JAK)-signal transducer and activator of transcription (STAT) signaling, known to be involved in DLBLC [18, 45, 46]. Lastly, Ujj et al. showed that higher levels of the Wilms tumor 1 (WT1) mRNA were associated with worse disease-free survival (DFS) and OS. WT1 is a DNA-binding protein with a complex function due to its many isoforms. Although WT1 is known to act as a tumor suppressor in Wilms tumor, it seems to be an oncogene in other neoplasms [47].

**Table 1:** Cell-free mRNA in DLBCL

References	Method	Sample	mRNA	Level	Result
*Diagnosis*					
Garcia et al. [41]	RT-qPCR plasma	42 DLBCL 50 controls	CCND2, BCL2, MYC, LMO2 BCL6, FN1	Up NS	Higher level in DLBCL No difference
Zhao et al.[44]	RT-qPCR peripheral blood	63 DLBCL 32 controls	CREBBP	Up	Higher level in DLBCL
*Response to therapy*					
Garcia et al. [41]	RT-qPCR plasma	42 DLBCL 50 controls	MYC CCND2, BCL2, LMO2, BCL6,FN1	Up NS	Higher level of MYC was associated with PR in patients with low-risk IPI No significant association with response to R-CHOP
Attia et al. [45]	RT-qPCR plasma	30 NHL (15 DLBCL) 20 controls	SOCS-3	Up	Higher level was associated with poor response to treatment (NR/PR versus CR) in NHL
*Prognosis*					
Garcia et al. [41]	RT-qPCR plasma	42 DLBCL 50 controls	MYC, CCND2 CCND2, BCL2, MYC	Up Up	Higher level of MYC or CCND2 was associated with worse OS, with the latter only in low-risk IPI group Higher level of CCND2, BCL2 or MYC in patients with CR was associated with worse PFS
Ujj et al. [47]	RT-qPCR PAXgene blood RNA tube	25 DLBCL 35 controls	WT1	Up	Higher level in pre-, intra- or posttreatment samples was associated with worse DFS and OS

*CR* complete response, *DFS* disease-free survival, *IPI* international prognostic index, *mRNA* messenger RNA, *NHL* non-Hodgkin’s lymphoma, *NR* no response, *NS* not significant, *OS* overall survival, *PFS* progression-free survival, *PR* partial response, *RT-qPCR* reverse-transcription quantitative polymerase chain reaction

The number of studies that have investigated cell-free mRNA is limited and the sample sizes are small. Moreover, none of the mRNAs have been studied by two or more different studies. Therefore, no well-founded conclusion can be made of their potential use and more studies are needed to validate these results. Compared to other types of RNA, research of circulating mRNA is challenging due to its relatively low abundance, as well as intracellular mRNA contamination and susceptibility to degradation [23, 48]. Despite these challenges, cell-free mRNAs may provide valuable insight in critical intracellular processes, both in tumor cells and in the cancer-associated microenvironment, as they possess a critical role in protein translation.

### MicroRNA

MicroRNAs (miRNAs) are short non-coding RNAs of ∼ 22 nucleotides, which are found in all eukaryotic cells. MiRNAs play pivotal roles in almost all biological pathways, regulating gene expression by targeting mRNA at the 3′-untranslated region [49, 50]. Since miRNAs can target up to several hundred mRNAs, aberrant expression can influence a multitude of cell signaling pathways, including cancer onset and progression. Contrary to mRNAs, miRNAs are resistant to ribonuclease digestion due to their small size and remain stable after being subjected to harsh conditions under which most other RNA would degrade [51]. Furthermore, studies have revealed genetic exchange between cells using miRNA in extracellular vesicles, such as exomes [26, 52]. MiRNAs are the most widely investigated subgroup of non-coding RNA in DLBCL. The first study investigating circulating miRNA in DLBCL was reported in 2008 by Lawrie et al., who demonstrated that serum levels of miR-21 were higher in DLBCL and were associated with relapse‐free survival (RFS) [53]. Since then, several cell-free miRNA have been proposed as biomarker [53] (Table 2).

**Table 2:** Cell-free miRNA in DLBCL

References	Method	Sample	miRNA	Level	Result
*Diagnosis*					
Lawrie et al. [53]	RT-qPCR serum	60 DLBCL 43 controls	miR-21, miR-155, miR-210	Up	Higher level in DLBCL
Fang et al. [55]	RT-qPCR serum	75 DLBCL 77 controls	miR-15a, miR-16-1, miR-29c, miR-155 miR-34a miR-21, miR-223	Up Down NS	Higher level in DLBCL Lower level in DLBCL No difference
Chen et al. [59]	RT-qPCR serum	62 DLBCL 50 controls	miR-21	Up	Higher level in DLBCL, especially in lower stage disease
Inada et al. [64]	RT-qPCR (exosome-enriched) serum	33 DLBCL 22 controls	miR-15a-3p, miR-21-5p, miR-210-5p miR-181a-5p miR-155-5p, miR-210-3p	Up Down NS	Higher level in DLBCL Lower level in DLBCL No difference
Li et al. [57]	RT-qPCR serum	112 DLBCL 45 controls	miR-21	Up	Higher level in DLBCL
Borges et al. [ 56]	RT-qPCR serum	21 DLBCL 6 controls	miR-17, miR-20b, miR-210, miR-296	NS	No difference
Yuan et al. [58]	RT-qPCR serum	56 DLBCL 20 controls	miR-21, miR-125b, miR-130a, miR-155, miR-200c miR-29c, miR-145, miR-451	Up Down	Higher level in DLBCL Lower level in DLBCL
Khare et al.[63]	RNA seq (exosome-enriched) plasma	14 DLBCL 20 controls	miR-124, miR-532-5p miR-122, miR-128, miR-141, miR-145, miR-197, miR-345, miR-424, miR-425, miR-101, let-7e, miR-222, miR-29c, miR-375, miR-324-5p, miR-135a, miR-379, let-7 i, miR-32	Up Down	Higher level in DLBCL Lower level in DLBCL
Bedewey et al. [79]	RT-qPCR serum	54 DLBCL 15 controls	miR-155	Up	Higher level in DLBCL
Zheng et al.[61]	RT-qPCR serum	203 DLBCL 100 controls	miR-21	Up	Higher level in DLBCL
Ahmadvand et al. [78]	RT-qPCR plasma	40 DLBCL 38 controls	miR-155	Up	Higher level in DLBCL
Meng et al.[62]	RNA seq/RT-qPCR serum	3 DLBCL 3 controls	miR-34a-5p miR-323b-3p, miR-431-5p 2588 miRNAs (51 different expressed miR)	Up Down	Higher level in DLBCL Lower level in DLBCL
Cui et al. [65]	RT-qPCR plasma	22 DLBCL 14 controls	miR-494, miR-21 mir-155	Up NS	Higher level in DLBCL No difference
Marchesi et al. [171]	Microarray/RT-qPCR serum	36 DLBCL 36 controls	miR-22, miR-18a, miR-20a, let-7c, miR-99a, miR-125b	NS	No difference
Zheng et al.[60]	RT-qPCR serum	200 DLBCL 100 controls	miR-155	Up	Higher level in DLBCL
Beheshti et al. [69]	ddPCR serum	86 DLBCL 17 controls	miR-15a, miR-18a, miR-24, let-7b, let-7c miR-10b, miR-27a, miR-130a, miR-155	Up NS	5-miRNA signature (higher level of let-7b, let-7c, miR-18a, miR-24, and miR-15a) was associated with DLBLC
Jorgensen et al. [66]	RT-qPCR plasma	38 DLBCL 41 controls	miR-199a-5p, miR-326, miR-328, miR-21-5p miR-375 miR 155-5p, miR-19a-3p, miR-19b-3p, miR-92a-3p, miR-10b-5p, miR-210, miR-363-3p	Up Down NS	Higher level in DLBCL Lower level in DLBCL Higher level of miR-199a-5p and miR-326, and lower level of miR-375 may predict future B-cell lymphoma development
*Subtype classification*					
Chen et al. [59]	RT-qPCR serum	62 DLBCL 50 controls	miR-21	Up	Higher level in ABC subtype compared to GCB subtype
Song et al. [81]	RT-qPCR serum	173 DLBCL	miR-33a, miR-224, miR-455-3p, miR-520d-3p, miR-1236	NS	No difference between ABC and GCB subtype
Bedewey et al. [79]	RT-qPCR serum	54 DLBCL 15 controls	miR-155	Up	Higher level in non-GCB compared to GCB subtype
*Response to therapy (R-CHOP)*					
Song et al. [81]	RT-qPCR serum	133 DLBCL	miR-224, miR-520d-3p, miR-1236 miR-33a, miR-455-3p Others (731 miRNA), including miR-21	Up Down NS	5-miRNA signature (higher level of miR-224, miR-520d-3p, miR-1236; lower level of miR-33a, miR-455-3p) was associated with superior response to therapy
Yuan et al. [58]	RT-qPCR serum	56 DLBCL 20 controls	miR-125b, miR-130a miR-21, miR-29c, miR-145, miR-155, miR-200c, miR-451	Up NS	Higher level was associated with inferior response. no association with response to therapy
Bouvy et al. [80]	Microarray/RT-qPCR plasma	19 DLBCL 1 control	miR-21, miR-197, miR-19b, miR-20a, miR-451 miR-122, let-7e	Up NS	Higher level of miR-21 and miR-197 during treatment was associated with inferior response. higher level of miR-19b, miR-20a, and miR-451 after 4 cycles R-CHOP differentiates patient group with CR from patient group with residual tumor
Cui et al. [65]	RT-qPCR plasma	56 DLBCL	miR-494, miR-21	Down	Higher pretreatment level of miR-494 was associated with interim-PET/CT negativity. Lower posttreatment miR-494 and miR-21 level compared to pretreatment values was associated with interim-PET/CT negativity
Marchesi et al. [171]	Microarray/RT-qPCR serum	36 DLBCL 36 controls	miR-22, let-7c, miR-125b, miR-99a	NS	No association with response to therapy
Fajardo-Ramirez et al. [82]	Microarray plasma	16 DLBCL	miR-105-5p, miR-186-5p, miR-19a-3p, miR-572, miR-1267, miR-555, miR-205-5p, miR-490-5p, miR-520d-3p miR-100-5p, miR-1910-5p, miR-24-3p, miR-628-3p, miR-766-3p, miR-615-3p	Up Down	15-miRNA signature differentiates patients with non-response from patients with CR
*Prognosis*					
Lawrie et al. [53]	RT-qPCR serum	52 DLBCL	miR-21 miR-155, miR-210	Up NS	Higher level was associated with favorable RFS No association with prognosis
Chen et al. [59]	RT-qPCR serum	62 DLBCL 50 controls	miR-21	Up	Higher level was associated with favorable RFS
Song et al. [81]	RT-qPCR serum	133 DLBCL	miR-224, miR-520d-3p, miR-1236 miR-33a, miR-455-3p 731 other miRNAs, including miR-21	Up Down NS	Higher level of miR-224, miR-520d-3p, miR-1236 and lower level of miR-33a, miR-455-3p was associated with lower MRT and higher probability of remission, independent of IPI score
Li et al. [57]	RT-qPCR serum	112 DLBCL 45 controls	miR-21	Up	Higher level was associated with inferior OS, independent of IPI score
Borges et al. [56]	RT-qPCR serum	21 DLBCL 6 controls	miR-17, miR-20b, miR-210, miR-296	NS	No difference in relapsed patients
Yuan et al. [58]	RT-qPCR serum	56 DLBCL 20 controls	miR-125b miR-130a	Up NS	Higher level was associated with inferior OS, independent of IPI score No significant association with OS
Bedewey et al. [79]	RT-qPCR serum	54 DLBCL 15 controls	miR-155	Up	Higher level was associated with inferior EFS
Marchesi et al. [171]	Microarray/RT-qPCR serum	36 DLBCL 36 controls	miR-22 miR-18a, miR-20a, let-7c, miR-99a, miR-125b	Up NS	Higher level was associated with inferior PFS after R-CHOP, independent of IPI score
Ahmadvand et al. [78]	RT-qPCR plasma	40 DLBCL 38 controls	miR-155	Up	Higher level was associated with inferior OS
Bouvy et al. [80]	Microarray/RT-qPCR plasma	19 DLBCL 1 control	miR-21, miR-197, miR-19b, miR-20a, miR-451	NS	No association with prognosis
Zheng et al.[60]	RT-qPCR serum	200 DLBCL 100 controls	miR-155	Up	Higher level was associated with inferior PFS following R-CHOP, independent of IPI
Sun et al. [84]	Microarray/RT-qPCR serum	120 DLBCL	miR-21, miR-130b, miR-155 miR-28	Up Down	4-miRNA signature (higher level of miR-21, miR-130b, miR-155; lower level of miR-28) was associated with relapse, as well as inferior PFS and OS after R-CHOP, independent of IPI score

*ABC* activated B-cell-like, *CR* complete response, *DFS* disease free survival, *ddPCR* droplet digital PCR, *DLBCL* diffuse large B-cell lymphoma, *EFS* event-free survival, *GCB* germinal center B-cell-like, *IPI* international prognostic index, *miR/miRNA* microRNA, *MRT* median remission time, *NR* no response, *NS* not significant, *OS* overall survival, *PFS* progression-free survival, *PR* partial response, *RFS* relapse-free survival, *RT-qPCR* reverse-transcription quantitative polymerase chain reaction

As diagnostic biomarkers in DLBCL, a total of eight different miRNAs (miR-15a, miR-21, miR-29c, miR-34a, miR-145, miR-155, miR-210, and miR-375) were found to be significantly dysregulated in at least two different studies. The studies concerning miR-145, miR-375, miR-15a, miR-21 and miR-155 presented the most concordant results, the first two miRNAs being lower and the following three being higher in DLBCL [53, 55–65]. MiR-145 and miR-375 were lower in two studies and are considered tumor suppressors in different cancer types. MiR-145 targets among others the oncogenes MYC and protein C-ets-1 (ETS1), and miR-375 targets the astrocyte elevated gene 1 (AEG-1), yes-associated protein 1 (YAP1), insulin like growth factor 1 (IGF1R) and pyruvate dehydrogenase kinase isoenzyme 1 (PDK1) [58, 63, 66–68]. MiR-15a was higher in three different studies [55, 64, 69]. Although being described as a tumor suppressor in CLL by targeting BCL-2, miR-15a has also been shown to target p53 in a miRNA/p53 feedback circuitry. Its higher abundance and role in DLBCL, however, remain unclear [70–72]. MiR-21 was higher in eight out of nine studies and one study showed no significant change. MiR-21 is considered to be an oncomiR that can be transcriptionally activated by nuclear factor kappa-light-chain-enhancer of activated B cells (NF-κB) and downregulates different phosphatases, such as programmed cell death protein 4 (PDCD4) and phosphatase and tensin homolog (PTEN), which are major players in crucial signaling pathways such as PI3K/AKT and mitogen-activated protein kinase (MAPK) [53, 55, 57–59, 61, 64–66]. Although its physiological function during normal B-cell development remains unclear, miR-21 is upregulated in GC and memory B cells compared to naive B cells and downregulated in plasma cells, which suggests a functional significance during differentiation. The decreased expression in plasma cells correlates with increasing B lymphocyte-induced maturation protein-1 (BLIMP-1) expression, the regulator of plasma cell differentiation [73, 74]. A mouse model demonstrated that miR-21 overexpression led to a pre-B malignant lymphoid-like phenotype and impacted the different stages of tumor development in vivo. Subsequent inactivation led to complete tumor regression mediated by increased apoptosis and proliferative arrest. This dependence may suggest an ‘oncogene addiction’ and therefore therapeutic potential of miR-21 inhibition [75]. Moreover, a positive feedback loop of sustained miR-21 upregulation and NF-κB activation has been demonstrated in non-DLBCL cancer cell lines, triggered by an inflammatory response and supporting oncogenic transformation [76]. These results warrant further exploration of miR-21 in DLBCL. MiR-155 was higher in six out of ten studies and four studies showed no significant change. MiR-155 is generally known as an oncomiR in many different tumors, targeting genes that play a central role in lymphomagenesis, such as suppressor of cytokine signaling (SOCS), phosphatidylinositol 3-kinase regulatory subunit alpha (PIK3R1) and src homology 2-domain-containing inositol 5′-phosphatase 1 (SHIP1) [53, 55, 58, 60, 64–66, 69, 77–79]. Lastly, Beheshti et al. combined five miRNAs in a signature that was able to differentiate DLBCL patients from healthy controls with a classification rate of 91% [69]. To this date, miR-155 and especially miR-21 seem to show the most promising potential as diagnostic biomarker. These findings are in line with a recent systematic review regarding miRNA analysis on tissue specimens [77]. Interestingly, dysregulation of cell-free miRNAs may precede the diagnosis up to 2 years, highlighting their potential as early biomarkers, before the onset of clinical symptoms [66].

For DLBCL subclassification, no miRNA was found to be significantly dysregulated in at least two different studies. MiR-21 and miR-155 expression levels were higher in the ABC subgroup in two smaller studies [59, 79]. Since these observations have not been validated, their use as biomarker remains uncertain. On tissue specimens, the most consistent results were obtained for miR-155-5p and miR-221-3p, which were upregulated in the ABC subgroup. These miRNAs may function through repression of PIK3R1, activating the PI3K/AKT signaling pathway [77].

In predicting response to R-CHOP treatment, miR-21 and miR-520 were found to be significantly dysregulated in at least two different studies. MiR-21 was investigated by four different studies. Higher levels during treatment were associated with inferior response in one study [80], decreasing posttreatment levels compared to pretreatment levels with superior response in another study [65] and no association was found in two other studies [58, 81]. In the study of Cui et al., although pretreatment levels were not associated with interim PET-CT status, the individual kinetics of mir-21 were associated with therapy response with decreasing values in patients that obtain interim PET-CT negativity in comparison to stable values in patients that remain PET-CT positive [65]. Two studies developed a 5- and 15-miR signature respectively, associated with response to R-CHOP therapy. Although miR-520 was higher in both signatures, it was associated with response in one study and non-response in the other [81, 82]. A meta-analysis conducted on tissue samples demonstrated a significant association between aberrant expression of miR-155, miR-17/92 clusters, miR-21, miR-224 and miR-146b-5p and poor treatment outcomes [83]. None of these have been significantly associated with treatment response in two or more studies as circulating biomarker.

Concerning prognosis in DLBCL, only cell-free miR-21 and miR-155 have been studied in more than one study. The studies concerning miR-21 presented conflicting results, with two studies showing higher levels as an independent poor prognostic factor [57, 84], two studies demonstrating higher levels as a favorable prognostic factor [53, 59], and two studies finding no significant association [80, 81]. In the studies with a favorable prognosis, however, no multivariate analysis was performed and there was no association between miRNA levels and clinicopathological features. As a result, the significance of miR-21 in the prognosis of DLBCL remains uncertain. Of the five studies concerning miR-155, four showed an significant association with inferior prognosis [60, 78, 79, 84] and one study could not demonstrate a significant association [53]. MiR-155 has also been proposed as prognostic biomarker by several studies on DLBCL tissue samples, although no clear conclusion could be retained [77]. Zheng et al. showed that a higher level of miR-155 may be associated with lymphoma progression through modulating PD-1/PD-L1-mediated interaction with CD8+ T-cells of tumor microenvironment, indicating sensitivity to PD-L1 blockade [60]. Lastly, two studies have proposed a signature of different miRNAs as prognostic predictor, independent of IPI score. Song et al. developed a 5-miRNA signature, associated with median remission time and probability of remission [81]. Another 4-miRNA prognostic model was significantly associated with relapse, as well as with worse PFS and OS [84].

Although there are some conflicting data, circulating miR-155 and especially miR-21 seem to hold the greatest potential as biomarkers for the diagnosis and subclassification of DLBCL. Regarding the prediction of therapy response, there is currently insufficient evidence for each of the proposed miRNA candidates. Concerning prediction of prognosis, miR-155 has been associated with inferior prognosis in several independent studies, although validation is needed. Lastly, several signatures that combine multiple miRNAs show promising results in the diagnosis, prediction of therapy response and prognosis in DLBCL. Their potential should be further investigated in future studies with large sample sizes.

### Extracellular vesicles

Extracellular vesicles (EVs) are a heterogeneous group of membrane-bounded vesicles that play an important role in intercellular communications. EVs are secreted by presumably all cell types and transfer a wide range of molecules in their cargo including proteins, lipids, and nucleic acids, mirroring their cell of origin. Since their discovery by Wolf et al. in 1967, their roles in normal physiology and in various pathologies such as cancer have been the subject of intensive research [85–87]. Accumulating evidence shows that a heterogeneous population of cells may communicate via EVs, hereby mediating processes involved in tumorigenesis. EVs are mostly classified as exosomes, microvesicles (MV) or apoptotic bodies (AB) on the basis of their size, origin, and characteristics [88]. Exosomes are vesicles of endosomal origin with a size ranging between 40 and 150 nm. Their formation consists of several steps, starting with endocytic vesicle invagination on the plasma membrane, followed by the creation of multiple intraluminal vesicles within the endosome via the folding of its phospholipid bilayer, and eventually the fusion of this multi-vesicular body with the plasma membrane to release exosomes [89, 90]. MVs are larger and more heterogeneous in size, ranging from 100 nm to several microns, are produced as a result of bulging of the plasma membrane, and are shed from the cell surface as these blebs undergo fission upon stimulation. ABs are the largest particles, ranging in size from 800 to 5000 nm, which are produced during cellular blebbing and released by cells undergoing programmed cell death [91].

Exosomes are the best characterized EVs and are found in most biofluids, such as blood, serum, urine, cerebral spinal fluid, and even breast milk [90, 92]. An abundant number of exosomes is released by tumor cells in comparison with non-tumorigenic cells [93]. They function as mediators of intercellular communication by transferring their content into other recipient cells utilizing various mechanisms, such as macropinocytosis, receptor or lipid raft mediated endocytosis, phagocytosis, or direct fusion with the recipient cell membrane. Exosomes have been shown to play a pivotal role in normal physiology and disease, including maintenance of cellular homeostasis, regulation of gene transcription, activation and modulation of immune response and cancer progression [90, 92, 94] Several studies have examined exosome-derived RNA as potential biomarker in DLBCL (Table 3).

**Table 3:** Extracellular vesicle-derived RNA in DLBCL

References	Method	EV purification (QC)	Sample	RNA	Level	Result
*Diagnosis*						
Caivano et al. [172]	RT-qPCR serum	DC (AFM/TEM)	5 DLBCL 18 controls	miR-155	NS	No difference
Provencio et al. [103]	RT-qPCR plasma	DC (ACHE activity)	60 DLBCL/38 FL 68 controls	BCL6 mRNA PTEN mRNA	Up Down	Higher level in mixed population of DBLCL/FL Lower level in mixed population of DBLCL/FL
Di et al. [96]	Micro-array/ RT-qPCR serum	ExoQuick (NTA/TEM/WB)	99 DLBCL 94 controls	miR-379-5p, miR-135a-3p, miR-4476 miR-483-3p, miR- 451a	Up Down	5-miRNA signature (higher level of miR-379-5p, miR-135a-3p, miR-4476; lower level of miR-483-3p, miR-451a) was associated with DLBCL
Xiao et al. [95]	RT-qPCR serum	ExoQuick (DLS/TEM/WB)	89 DLBCL 48 controls	miR-451a	Down	Lower level in DLBCL
Zare et al. [173]	RT-qPCR plasma	Exo-spin (Zetasizer/TEM/DB/ WB)	48 DLBCL 6 controls	miR-146a	NS	No difference
*Response to therapy*						
Provencio et al. [103]	RT-qPCR plasma	DC (ACHE activity)	16 DLBCL/15 FL 68 controls 60 DLBCL/38 FL 68 controls	BCL6 mRNA PTEN mRNA	Up Down	Higher level of BCL-6 in posttreatment samples compared to pretreatment samples was associated with NR in mixed population of DBLCL/FL. lower level of PTEN at time of diagnosis was associated with PD/R in DLBCL subgroup
Feng et al. [102]	RNA seq/RT-qPCR/serum	ExoQuick (DLS/ TEM/WB)	116 DLBCL	miR-99a-5p, miR-125b-5p miR-10a-5p, miR-10b-5p	Up NS	Higher level differentiates therapy resistant group (SD/PD) from sensitive group (CR/PR) after R-CHOP
Zare et al. [173]	RT-qPCR plasma	Exo-spin (Zetasizer/TEM/DB/WB)	48 DLBCL 6 controls	miR-146a	NS	No association with treatment response after R-CHOP
Xiao et al. [95]	RT-qPCR serum	ExoQuick (DLS/TEM/WB)	89 DLBCL 48 controls	miR-451a	Up	Higher level differentiates response group (PR/CR) from non-response group (SD/PD) after R-CHOP
Zare et al. [101]	RT-qPCR plasma	Exo-spin (Zetasizer/TEM/WB)	48 ABC DLBCL	miR-155 miR-let-7 g miR-let-7i	Up Down NS	Higher level of miR-155 in non-responsive (PD/SD) and relapsed patients compared to responsive patients (CR/PR) and patients receiving R-CHOP Lower level of miR-let-7 g in patients receiving R-CHOP compared to non-responsive (PD/SD) and relapsed patients
*Prognosis*						
Provencio et al. [103]	RT-qPCR plasma	DC (ACHE activity)	16 DLBCL/15 FL 68 controls 60 DLBCL/38 FL 68 controls	BCL-xL mRNA AKT, C-MYC, BCL-6 mRNA	Up Up	Higher posttreatment level of BCL-xL was associated with a higher death rate in mixed population of DBLCL/FL Pretreatment presence of AKT and C-MYC/BCL-6 was respectively associated with worse PFS and OS in a mixed population of DBLCL/FL patients, responsive to rituximab-based chemotherapy
Feng et al. [102]	RNA seq/RT-qPCR/serum	ExoQuick (DLS/ TEM/WB)	116 DLBCL	miR-99a-5p, miR-125b-5p	Up	Higher level is associated with worse PFS

*ABC* activated B-cell-like, *ACHE* acetylcholinesterase, *AFM* atomic force microscopy, *CR* complete response, *DB* dot blot, *DC* differential centrifugation, *DFS* disease-free survival, *DLS* dynamic lights scattering, *DLBCL* diffuse large B-cell lymphoma, *EFS* event-free survival, *GCB* germinal center B-cell-like, *IPI* international prognostic index, *mRNA* messenger RNA, *miR* microRNA, *NR* no response, *NS* not significant, *NTA* nano particle tracking analysis, *OS* overall survival, *PFS* progression-free survival, *PD* progressive disease, *PR* partial response, *QC* quality control, *R* relaps; *RT-qPCR* reverse-transcription quantitative polymerase chain reaction, *TEM* transmission electron microscope

Concerning diagnosis and classification, only exosomal miR-451 was significantly dysregulated in at least two different studies [95, 96]. MiR-451 is considered a tumor suppressor in different cancers. Although its role in DLBCL remains unclear, targets include c-MYC and the PI3K/AKT pathway [97, 98]. Di et al. identified an exosome-derived 5-miRNA signature that could differentiate DLBCL patients from healthy controls, which included lower levels of miR-451 [96]. Using DLBCL cell lines, Rutherford et al. showed that nearly one third of mutations in DLBCL are detectable in the EVs and that exosomal RNA may to reflect the cell of origin [99, 100]. However, to this date, there are no studies that could distinguish ABC and GCB subtypes using exosome-derived RNA in DLBCL patients.

Regarding therapy response, none of the biomarkers were examined in two different studies. Exosome-derived miR-99a-5p, miR-125b-5p, miR-155 and miR-451a were associated with therapy response in single studies [95, 101, 102]. Provencio et al. showed that increase of BCL-6 mRNA in posttreatment samples compared to pretreatment samples was associated with non-response to therapy in a mixed population of DBLCL and FL patients. Furthermore, lower abundance of PTEN at time of diagnosis was associated with PD/R in the DLBCL subgroup [103]. Interestingly, using DLBCL cell lines, Koch et al. demonstrated a mechanism of exosome-mediated removal of the anthracycline doxorubicin and the anthracenedione pixantrone from the cell nucleus, associated with decreased therapy efficiency. They showed that inhibition of ATP-transporter A3 (ABCA3), a protein involved in exosomal transport, resulted in higher sensitivity to these drugs [104]. The inhibition of ABCA3 has also been shown to play a role in creating decreased sensitivity to anti-CD20 immunotherapy through the upregulation of exosomal CD20 [105].

Concerning prognosis, higher levels of exosomal miR-99a-5p and miR-125b-5p have been associated with inferior PFS in a single studie [102]. Provencio et al. showed that pretreatment presence of exosome-derived AKT and C-MYC/BCL-6 was respectively associated with worse PFS and worse OS in a mixed population of responsive DBLCL/FL patients [103]. Since none of the biomarkers were examined in two different studies, no well-grounded conclusions can be made concerning their potential use in prediction of prognosis.

Exosomes have been shown to interact with the immune system. In vitro uptake of DBLCL cell line-derived exosomes in B-cells and monocytes has been demonstrated, while T-cells and NK cells displayed significantly lower uptake [106]. Exosomes contain tumor antigens, as well as MHC class I/II molecules, thus allowing direct activation of and cross-presentation to T-cells [107]. Zare et al. demonstrated adverse effect of plasma-derived exosomes of DLBCL patients on several functions of NK cells [108]. Furthermore, tumor-derived exosomes play a role in immune invasion by secretion of PD-L1, thereby suppressing T-cell activation. Poggio et al. showed that local blockade of exosomal PD-L1 inhibited both local and distant tumoral growth, even in models resistant to anti-PD-L1 antibodies [109]. Similarly, Chen et al. showed that exosomes act as immunosuppressive mediators, evidenced by an upregulation of PD-1 and induction of apoptosis in T-cells. Furthermore, the exosomes enhanced cell proliferation, invasion, migration, and angiogenesis, promoting tumor growth in vivo [110]. Koch et al. demonstrated the presence of “side population cells” in DLBCL, which have stemness properties and are capable of propagating tumor growth. These cells are in equilibrium with their environment, regulated through exosome-mediated Wingless-related integration site (Wnt) signaling [111].

There are several important pitfalls in the analysis of tumor-derived exosomes. EVs are usually classified on the basis of their origin. However, this classification and current techniques are insufficient to clearly distinguish each type separately. There are major differences between used protocols with the accuracy and the purity of preparations being highly dependent on the isolation method used by different laboratories, leading to discrepancies in the EV subpopulation obtained [112–114]. There is need for a standardized approach, such as proposed by the Minimal Information for Studies of Extracellular Vesicles (“MISEV”) guidelines, which provide protocols and procedures to minimize interlaboratory variation [88]. Another challenge is the need to enrich for tumor-specific exosomes in order to reduce background noise from non-neoplastic-derived exosomes. Furthermore, studies in DLBCL show conflicting data whether tumor-derived RNA is enriched in exosomes compared to serum/plasma [63–65, 96, 101]. In other cancers, antibodies to specific surface proteins have been used to isolate the tumor-derived exosomes [115]. Alternatively, bioinformatic deconvolution techniques can be used to differentiate tumor-derived signals from normal and neoplastic tissue. The use of disease-specific EV-derived markers in DLBCL has promising potential, but further research is needed in a more standardized way to allow direct comparison of studies.

### Long non‐coding RNA

Approximately 80% of the human genome is non-coding, but functional. Long non-coding RNA (lncRNA) comprises a large and heterogeneous class of transcripts, arbitrarily defined as being more than 200 nucleotides in length [116]. They are located within intergenic, intronic, or antisense stretches or they overlap with protein-coding genes [117]. LncRNAs are expressed in different human biofluids and are resistant to degradation by ribonucleases, being encapsulated inside EVs or in association with proteins [118]. They are involved in essential processes, such as chromatin remodeling, transcriptional regulation, and posttranscriptional modification [119]. LncRNA expression profiling during B-cell development has been performed in several studies that report cell type-specific expression patterns at various stages of B-cell development [120–122]. It has also been suggested that lncRNAs may play a role in the chromosome breaks involved in typical gene rearrangements in hematologic malignancies, such as BCL-6 translocation [123].

The vast majority of studies concerning dysregulation of lncRNA in DLBCL have been performed on tissue samples. Verma et al. examined large RNA-seq data sets from 116 DLBCL tissue samples and identified 2632 novel lncRNAs, most of which were only expressed in malignant cells. Interestingly, more than one third of these lncRNAs were differently expressed between the GCB and ABC subtypes [124]. Sun et al. analyzed lncRNA expression profiles in three cohorts of 1043 DLBCL patients using microarray data from the Gene Expression Omnibus (GEO) database. They identified and validated a 6-lncRNA signature that was significantly associated with OS, independent of conventional clinical factors [125]. Zhou et al. reanalyzed the data sets and reported a 17-lncRNA signature, that was significantly associated with OS and PFS, but that was also able to distinguish between COO subtypes with over 90% accuracy [126]. Gao et al. developed a 5-lncRNA signature that was differentially expressed between GCB-DLBCL tissue samples compared to reactive lymph nodes [127]. Lastly, an in silico analysis analyzed 189 lncRNAs, extracted from the HUGO Gene Nomenclature Committee (HGNC), and demonstrated that 83% of DLBCL patients showed dysregulation for the studied lncRNAs. Growth arrest-specific 5 (GAS5), highly up-regulated in liver cancer (HULC), miR-17-92a-1 cluster host gene (MIR17HG), and prostate cancer antigen 3 (PCA3) were the transcripts with the highest dysregulation score [128]. The potential of lncRNA expression profiling as biomarker in DLBCL was underlined by a meta-analysis conducted by Xu et al. [129].

The biological relevance of circulating ncRNAs is becoming increasingly clear, as their diagnostic and prognostic value has been shown in several solid tumors, such as bladder, colorectal, esophageal, prostate, hepatocellular, cervical, breast, gastric and non-small-cell lung cancer [130]. The group of Isin et al. were the first to investigate circulating lncRNA in B-cell malignancies, more specifically in CLL and multiple myeloma. They showed a dysregulation of plasma lincRNA-p21 levels in CLL patients and taurine upregulated gene 1 (TUG1), metastasis associated lung adenocarcinoma transcript 1 (MALAT1), HOX transcript antisense RNA (HOTAIR) and GAS5 in multiple myeloma patients [131]. Wang et al. are the only group to investigate circulating lncRNAs in DLBCL, demonstrating lower levels of p21 associated ncRNA DNA damage activated (PANDA) and higher abundance of TUG1 in DLBCL patients, compared to healthy controls (Table 4). Moreover, lower levels of PANDA were associated with inferior RFS and OS, independent of disease stage. They showed that PANDA was induced by p53 and can suppress cell proliferation through inactivation of MAPK signaling pathway. The same group analyzed the expression of lncRNAs in DLBCL tissue samples and found an upregulation of TUG1, HULC, HOTAIR, and a downregulation of PANDA, lincRNA-p21 and FLJ46300 in DLBCL, with tissue-derived PANDA also being associated with OS [132].

**Table 4:** Cell-free lncRNA in DLBCL

References	Method	Sample	lncRNA	Level	Result
*Diagnosis*					
Wang et al. [132]	RT-qPCR serum	68 DLBCL 68 controls	PANDA TUG1 HOTAIR, HULC, FLJ46300, lincRNA-p21	Down Up NS	Lower level in DLBCL Higher level in DLBCL No difference
*Prognosis*					
Wang et al. [132]	RT-qPCR serum	68 DLBCL 68 controls	PANDA	Down	Lower level was associated with worse RFS and OS, independent of disease stage

*DLBCL* diffuse large B-cell lymphoma, *lncRNA* long non-coding RNA, *NS* not significant, *OS* overall survival, *RFS* relapse-free survival, *RT-qPCR* reverse-transcription quantitative polymerase chain reaction

Several individual lncRNA genes have been associated with DLBCL in tissue-derived specimens and could therefore be promising circulating biomarkers. LincRNA-p21 and NONHSAG026900 are shown to be downregulated in DLBCL tissue and are associated with favorable OS, as both lncRNA possibly act as tumor suppressors [133, 134]. Moreover, NONHSAG026900 was elevated in the GCB subtype compared to the non-GCB subtype [134]. Leukemia-associated non-coding IGF1R activator RNA 1 (LUNAR1), functional intergenic repeating RNA element (FIRRE), olfactory receptor family 3 subfamily A member 4 (OR3A4) and retrotransposon-derived protein PEG10 (PEG10) are upregulated in DLBCL tissue samples and associated with inferior OS, acting as oncogenes [135–138]. Peng et al. demonstrated an increased HULC expression in DLBCL samples and higher levels were independently associated with inferior OS/PFS [139]. MALAT1, a lncRNA that is upregulated in different solid and hematological tumors and that has been associated with cancer metastasis and recurrence [131, 140, 141], seems to be involved in chemotherapy sensitivity in DLBCL cell lines by enhancing autophagy-related proteins [142]. HOTAIR is a well-known contributor to tumorigenesis [143]. Two studies reported conflicting results regarding HOTAIR expression and prognosis in DLBCL [144, 145]. The lncRNA nuclear para-speckle assembly transcript 1 (NEAT1) has also been associated with poor prognosis and may act as a competing endogenous RNA (ceRNA), regulating the miR-34b-5p-GLI1 axis, stimulating the proliferation of DLBCL [146, 147]. Lastly, several studies have shown aberrant expression patterns of the lncRNAs small nucleolar RNA host gene 12 (SNHG12), SNHG14, SNHG16, TUG1, MALAT1 and SMAD5 antisense RNA 1 (SMAD5-AS1) in DLBCL samples and cell lines, indicating their use as potential biomarkers, although further investigation is warranted [148–153]. Huang et al. recently reviewed functional studies of lncRNAs in DLBCL and their respective role in tumor cell biology [154].

Since several promising biomarkers have been proposed based on tissue samples, future research should focus on analyzing their expression in serum or plasma. Furthermore, since many lncRNAs have only been reported in a single study, there is a need for systematic validation studies that investigate multiple lncRNAs with well-characterized and diverse patient samples.

### Circular RNA

Circular RNAs (circRNAs) are a recently discovered subclass of large ncRNA, widely expressed in mammalian cells. They originate from a host gene and are formed through a backsplicing event, linking the 3′ end of an exon to the 5′ end of the same or an upstream exon [155]. CircRNAs may function as direct or indirect regulators of host gene expression at the transcriptional level, as protein scaffolds, as sponges of miRNAs, as regulators of protein translation, or under certain circumstances even as templates for translation [156, 157]. Many circRNAs are highly evolutionary conserved, resistant to exonucleases due to their closed structure, and thus more stable than linear non-coding RNAs such as miRNA and lncRNA, which highlights their potential to serve as cell-free biomarkers [158, 159].

No studies have yet examined their role during normal B-cell development and differentiation. However, specific circRNA signatures seem to be characteristic for B-cells compared to T-cells and progenitors [160]. Furthermore, circRNAs have been shown to play a role in the pathogenesis of cancer and circRNA expression profiles can differentiate between different B-cell malignancies [161, 162]. Several circRNAs are rich in miRNA-binding sites and can act as highly efficient ceRNA in cells by competing in binding to miRNAs, facilitating tumorigenesis. Using DLBCL cell lines, Chen et al. showed that circCFL1 targets the miR-107 target gene HMGB1, resulting in an increased expression of HMGB1, thereby upregulating the phosphorylation levels of p-AKT, p-ERK, and p-STAT3, which are involved in signaling pathways that control cell proliferation and migration [163]. Research in Burkitt lymphoma demonstrated a MYC-miR-150-ZDHHC11/B-MYB network, in which high levels of MYC repress miR-150, which leads to derepression of MYB, ZDHHC11 and ZDHHC11B, and promotes proliferation. Moreover, upregulation of the circRNAs ZDHHC11 and ZDHHC11B increases expression of the MYC target MYB proto-oncogene through a mechanism in which they could act as ceRNAs for miR-150 [164]. Yang et al. showed that circRNA circAmotl1 promotes nuclear translocation of MYC, as well as upregulation of MYC targets and, therefore, plays a possible role in lymphomagenesis [165]. Since MYC upregulation is frequently observed in DLBCL, these circRNAs may be of importance in further research. Interestingly, chromosomal translocations have been described producing fusion-circRNAs [166].

A recent study by Hu et al. is the first to explore the potential of circRNA as biomarker in DLBCL. They demonstrated that circAPC was significantly downregulated in DLBCL tissues and associated with aggressive clinical features and poor prognosis. CircAPC elevates the expression of the host gene adenomatous polyposis coli (APC), thereby inactivating canonical Wnt/β-catenin signaling and restraining DLBCL growth. Moreover, circAPC levels in plasma sample were also significantly lower in DLBCL patients and could serve as a potential diagnostic noninvasive marker [167] (Table 5). These results have to be confirmed in future studies.

**Table 5:** Cell-free circRNA in DLBCL

References	Method	Sample	circRNA	Level	Result
*Diagnosis*					
Hu et al. [167]	RT-qPCR plasma	27 DLBCL 16 controls	Circ-APC	Down	Lower level in DLBCL

*DLBCL* diffuse large B-cell lymphoma, *circRNA* circular RNA, *RT-qPCR* reverse-transcription quantitative polymerase chain reaction

Although circRNAs are increasingly being investigated in cancer research, there are several challenges. Firstly, there is a need for a common standard in reporting and naming circRNAs. Secondly, circRNAs lack poly(A) tails, which are often relied on for the purification step in order to remove ribosomal RNA during sequencing. Substantial methodological difficulties like template switching and rolling circle amplification during reverse transcription, as well as amplification bias during PCR have been formulated as well [161, 168]. Moreover, results depend highly on the strategy of library preparation and choice of one of the many available bioinformatic algorithms. Some of these challenges are, however, increasingly being encountered using newer techniques, such as the Nanopore RNA sequencing or the nCounter platform, showing promising potential to further explore the use of circRNAs as biomarkers in malignant diseases [161, 162].

### Tumor‐educated platelets

Blood platelets are small, circulating cell fragments that originate from megakaryocytes. They are best known for their role in hemostasis and initiation of wound healing. Platelets as part of the microenvironment, however, are also increasingly recognized to be involved in tumoral processes in which they stimulate proliferation, facilitate metastasis, and induce phenotypic changes in cancer cells. Because of their anucleate status, the majority of transcriptional production of RNA occurs in the megakaryocyte. Therefore, the RNA profile in platelets reflects the transcriptional status of megakaryocytes, as well as the bone marrow or other local environmental signals involved at the time of platelet production [29]. Increasing evidence shows that platelets can also be ‘educated’ by tumors (tumor-educated platelets; TEPs) by altering the platelets RNA profile, mainly through the process of specific pre-mRNA splicing and to lesser extent by ingestion of tumor-derived circulating mRNA [27–31]. These changes may be induced by cancer cells or by other external stimuli from the microenvironment [30]. Through these mechanisms, platelets have a rich repertoire of RNAs, including mRNAs, structural and catalytic RNAs (ribosomal RNA, transfer RNA, and small nucleolar RNA), and regulatory RNAs (microRNA and long non-coding RNA) [28, 29, 31, 169].

Best et al. showed that mRNA sequencing of TEPs discriminated metastatic cancer cases from healthy controls with 96% accuracy and localized primary tumors with 71% accuracy across six different solid tumor types. The tumor-specific educational programs in TEPs were predominantly influenced by tumor type, and to a lesser extent, by tumor progression and metastasis. The signatures showed widespread correlation with cancer tissues and a negative correlation with RNAs implicated in RNA translation, T-cell immunity, and interleukin signaling, the latter implying an important role in regulating the immune response in the tumoral microenvironment. Lastly, they identified several tumor-derived genetic alterations in TEPs [28]. In a recent study, the spliced RNA profile of TEPs was able to differentiate glioblastoma patients from healthy controls with an accuracy of 95%. Moreover, individual TEP tumor scores represented tumor behavior and were able to distinguish false positive progression from true progression with an accuracy of 85% [170]. These findings suggest that TEPs contain a unique source of functional and genomic tumoral information, obtained by a mechanism distinct from that which generates circulating-free DNA and RNA, which may implicate an complementary role in personalized non-invasive cancer management. At this time, no studies of TEPs have been performed in DLBCL, although this could produce a valuable contribution to the research field of liquid biopsy.

## Conclusion and future perspectives

In recent years, major progress has been made in identifying cell-free RNA biomarkers for the diagnosis, disease subtype classification, prediction of treatment response or prognosis in DLBCL. The vast majority of research has been performed in circulating (vesicle-encapsulated) miRNA, where some have shown consistent changes in several independent studies and could be further explored as diagnostic and prognostic biomarkers. While promising results were reported for different solid tumors, the use of cell-free mRNA, lncRNA, circRNA, and TEP-derived RNA has only sparsely been investigated in DLBCL and further studies are needed to analyze their potential.

There are no studies investigating the mutational profile, editing and alternative splicing of circulating RNA in DLBCL patients. We believe that further research should focus on other aspects beyond abundance, as this can provide valuable information on the cell of origin, as well as on the function of circulating RNAs. In the same vein, a promising future perspective is combining cell-free DNA and RNA, as well as other cellular molecules, in an multiomics approach. This could have far-reaching impact and push the field forward in identifying minimal invasive, disease-specific biomarkers that can be routinely implemented in clinical practice.

Concerning methodology, the development of unique circulating signatures in DLBCL with high specificity and sensitivity requires a standardized and consistent approach that must be applied during the whole research process, from blood collection to plasma/serum preparation, handling, and banking to extraction and quantification. Furthermore, defining and establishing validated reference sample sets is necessary. In addition, the experimental design of the study must be clear, including an adequate sample size relative to the objectives and possible variabilities, as well as validation of the results in an independent cohort, which is not included in most studies. Concerning the use of EV in biomarker development, a standardized workable isolation method is needed to ensure that the same term covers the same load in different research. Future progress in characterizing the content of lipid vesicles and unraveling the processes involved in their formation and function will contribute to a better understanding of their potential use as biomarkers in malignant diseases. Lastly, it would be important to see how cell-free biomarker candidates would perform in large prospective clinical trials.

## Data Availability

Data sharing is not applicable to this article as no datasets were generated or analysed.

## Abbreviations

AB: Apoptotic body
ABC: Activated B-cell-like
AEG-1: Astrocyte elevated gene 1
APC: Adenomatous polyposis coli
BCL-2/BCL-6/BCL-xL: B-cell lymphoma 2/B-cell lymphoma 6/B-cell lymphoma extra large
BLIMP-1: B lymphocyte-induced maturation protein-1
CCND2: G1/S-specific cyclin-D2
ceRNA: Competing endogenous RNA
cfDNA: Cell-free DNA
circRNA: Circular RNA
CLL: Chronic lymphatic leukemia
COO: Cell-of-origin
CR: Complete response
CREBBP: CREB binding protein
CTC: Circulating tumor cell
ctDNA: Circulating tumor DNA
DFS: Disease-free survival
DLBCL: Diffuse large B-cell lymphoma
ETS1: Protein C-ets-1
EV: Extracellular vesicle
FIRRE: Functional intergenic repeating RNA element
GAS5: Growth arrest-specific 5
GCB: Germinal center B-cell-like
GEP: Gene expression profiling
HMGB1: High mobility group box protein 1
HOTAIR: HOX transcript antisense RNA
HULC: Highly up-regulated in liver cancer
IGF1R: Insulin like growth factor 1 receptor
IPI: International prognostic index
JAK/STAT: Janus kinase/signal transducer and activator of transcription
LMO2: LIM domain only 2
lncRNA: Long non-coding RNA
LUNAR1: Leukemia-associated non-coding IGF1R activator RNA 1
MAGE-A3: Melanoma-associated antigen 3
MALAT1: Metastasis associated lung adenocarcinoma transcript 1
MAPK: Mitogen-activated protein kinase
miRNA/miR: MicroRNA
mRNA: Messenger RNA
MIR17HG: miR-17-92a-1 cluster host gene
MV: Microvesicle
MYB: MYB proto-oncogene
MYC: MYC proto-oncogene
NEAT1_1: Nuclear para-speckle assembly transcript 1_1
NF-κB: Nuclear factor kappa-light-chain-enhancer of activated B cells
NHL: Non-Hodgkin’s lymphoma
OR3A4: Olfactory receptor family 3 subfamily A member 4
OS: Overall survival
PANDA: p21 associated ncRNA DNA damage activated
PCA3: Prostate cancer antigen 3
PDCD4: Programmed cell death protein 4
PDK1: Pyruvate dehydrogenase kinase isoenzyme 1
PEG10: Retrotransposon-derived protein PEG10
PET-CT: Positron emission tomography–computed tomography.
PFS: Progression-free survival
PI3K/AKT/mTOR: Phosphatidylinositol 3/kinase-protein kinase B/mammalian target of rapamycin
PIK3R1: Phosphatidylinositol 3-kinase regulatory subunit alpha
PRC2: Polycomb repressive complex 2
PTEN: Phosphatase and tensin homolog
p21: Cyclin-dependent kinase inhibitor 1
p53: Tumor protein p53
R-CHOP: Rituximab, cyclophosphamide, vincristine, doxorubicin and prednisone
SHIP1: src homology 2-domain-containing inositol 5′-phosphatase 1
SMAD5-AS1: SMAD5 antisense RNA 1
SNHG12/SNHG14/SNHG16: Small nucleolar RNA host gene 12/small nucleolar RNA host gene 14/small nucleolar RNA host gene 16
SOCS: Suppressor of cytokine signaling
STAT3: Signal transducer and activator of transcription 3
TEP: Tumor-educated platelet
TUG1: Taurine upregulated gene 1
Wnt: Wingless-related integration site
YAP1: Yes-associated protein 1

## References

[1] Coiffier B, Lepage E, Briere J, Herbrecht R, Tilly H, Bouabdallah R (2002). CHOP chemotherapy plus rituximab compared with CHOP alone in elderly patients with diffuse large-B-cell lymphoma. N Engl J Med.

[2] Crump M, Neelapu SS, Farooq U, Van Den Neste E, Kuruvilla J, Westin J (2017). Outcomes in refractory diffuse large B-cell lymphoma: results from the international SCHOLAR-1 study. Blood.

[3] Farooq U, Maurer MJ, Thompson CA, Thanarajasingam G, Inwards DJ, Micallef I (2017). Clinical heterogeneity of diffuse large B cell lymphoma following failure of front-line immunochemotherapy. Br J Haematol.

[4] Rossi D, Diop F, Spaccarotella E, Monti S, Zanni M, Rasi S (2017). Diffuse large B-cell lymphoma genotyping on the liquid biopsy. Blood.

[5] Scherer F, Kurtz DM, Newman AM, Stehr H, Craig AF, Esfahani MS (2016). Distinct biological subtypes and patterns of genome evolution in lymphoma revealed by circulating tumor DNA. Sci Transl Med.

[6] Kurtz DM, Scherer F, Jin MC, Soo J, Craig AFM, Esfahani MS (2018). Circulating tumor DNA measurements as early outcome predictors in diffuse large B-cell lymphoma. J Clin Oncol.

[7] Hans CP, Weisenburger DD, Greiner TC, Gascoyne RD, Delabie J, Ott G (2004). Confirmation of the molecular classification of diffuse large B-cell lymphoma by immunohistochemistry using a tissue microarray. Blood.

[8] Alizadeh AA, Eisen MB, Davis RE, Ma C, Lossos IS, Rosenwald A (2000). Distinct types of diffuse large B-cell lymphoma identified by gene expression profiling. Nature.

[9] Schmitz R, Wright GW, Huang DW, Johnson CA, Phelan JD, Wang JQ (2018). Genetics and pathogenesis of diffuse large B-cell lymphoma. N Engl J Med.

[10] Chapuy B, Stewart C, Dunford AJ, Kim J, Kamburov A, Redd RA (2018). Molecular subtypes of diffuse large B cell lymphoma are associated with distinct pathogenic mechanisms and outcomes. Nat Med.

[11] Esfahani MS, Alig S, Kurtz DM, Soo J, Jin MC, Macaulay C (2019). Towards non-invasive classification of DLBCL genetic subtypes by Ctdna profiling. Blood.

[12] Alkodsi A, Cervera A, Zhang K, Louhimo R, Meriranta L, Pasanen A (2019). Distinct subtypes of diffuse large B-cell lymphoma defined by hypermutated genes. Leukemia.

[13] Lossos IS, Czerwinski DK, Alizadeh AA, Wechser MA, Tibshirani R, Botstein D (2004). Prediction of survival in diffuse large-B-cell lymphoma based on the expression of six genes. N Engl J Med.

[14] Shipp MA, Ross KN, Tamayo P, Weng AP, Kutok JL, Aguiar RC (2002). Diffuse large B-cell lymphoma outcome prediction by gene-expression profiling and supervised machine learning. Nat Med.

[15] Rosenwald A, Wright G, Chan WC, Connors JM, Campo E, Fisher RI (2002). The use of molecular profiling to predict survival after chemotherapy for diffuse large-B-cell lymphoma. N Engl J Med.

[16] International Non-Hodgkin’s Lymphoma Prognostic Factors P (1993). A predictive model for aggressive non-Hodgkin’s lymphoma. N Engl J Med.

[17] Wilson WH, Young RM, Schmitz R, Yang Y, Pittaluga S, Wright G (2015). Targeting B cell receptor signaling with ibrutinib in diffuse large B cell lymphoma. Nat Med.

[18] Hartert KT, Wenzl K, Krull JE, Manske M, Sarangi V, Asmann Y (2020). Targeting of inflammatory pathways with R2CHOP in high-risk DLBCL. Leukemia.

[19] Morschhauser F, Feugier P, Flinn IW, Gasiorowski R, Greil R, Illes A (2020). A phase II study of venetoclax plus R-CHOP as first-line treatment for patients with diffuse large B-cell lymphoma. Blood.

[20] Muringampurath-John D, Jaye DL, Flowers CR, Saxe D, Chen Z, Lechowicz MJ (2012). Characteristics and outcomes of diffuse large B-cell lymphoma presenting in leukaemic phase. Br J Haematol.

[21] Carninci P, Kasukawa T, Katayama S, Gough J, Frith MC, Maeda N (2005). The transcriptional landscape of the mammalian genome. Science.

[22] Esteller M (2011). Non-coding RNAs in human disease. Nat Rev Genet.

[23] Tsui NB, Ng EK, Lo YM (2002). Stability of endogenous and added RNA in blood specimens, serum, and plasma. Clin Chem.

[24] Ma R, Jiang T, Kang X (2012). Circulating microRNAs in cancer: origin, function and application. J Exp Clin Cancer Res.

[25] Sole C, Arnaiz E, Manterola L, Otaegui D, Lawrie CH (2019). The circulating transcriptome as a source of cancer liquid biopsy biomarkers. Semin Cancer Biol.

[26] Valadi H, Ekstrom K, Bossios A, Sjostrand M, Lee JJ, Lotvall JO (2007). Exosome-mediated transfer of mRNAs and microRNAs is a novel mechanism of genetic exchange between cells. Nat Cell Biol.

[27] Denis MM, Tolley ND, Bunting M, Schwertz H, Jiang H, Lindemann S (2005). Escaping the nuclear confines: signal-dependent pre-mRNA splicing in anucleate platelets. Cell.

[28] Best MG, Sol N, Kooi I, Tannous J, Westerman BA, Rustenburg F (2015). RNA-seq of tumor-educated platelets enables blood-based pan-cancer, multiclass, and molecular pathway cancer diagnostics. Cancer Cell.

[29] Schubert S, Weyrich AS, Rowley JW (2014). A tour through the transcriptional landscape of platelets. Blood.

[30] Kuznetsov HS, Marsh T, Markens BA, Castano Z, Greene-Colozzi A, Hay SA (2012). Identification of luminal breast cancers that establish a tumor-supportive macroenvironment defined by proangiogenic platelets and bone marrow-derived cells. Cancer Discov.

[31] McAllister SS, Weinberg RA (2014). The tumour-induced systemic environment as a critical regulator of cancer progression and metastasis. Nat Cell Biol.

[32] Schwarzenbach H, Hoon DS, Pantel K (2011). Cell-free nucleic acids as biomarkers in cancer patients. Nat Rev Cancer.

[33] Smith B, Selby P, Southgate J, Pittman K, Bradley C, Blair GE (1991). Detection of melanoma cells in peripheral blood by means of reverse transcriptase and polymerase chain reaction. Lancet.

[34] Kopreski MS, Benko FA, Kwak LW, Gocke CD (1999). Detection of tumor messenger RNA in the serum of patients with malignant melanoma. Clin Cancer Res.

[35] Souza MF, Kuasne H, Barros-Filho MC, Ciliao HL, Marchi FA, Fuganti PE (2017). Circulating mRNAs and miRNAs as candidate markers for the diagnosis and prognosis of prostate cancer. PLoS ONE.

[36] Silva J, Garcia V, Garcia JM, Pena C, Dominguez G, Diaz R (2007). Circulating Bmi-1 mRNA as a possible prognostic factor for advanced breast cancer patients. Breast cancer research: BCR.

[37] Pucciarelli S, Rampazzo E, Briarava M, Maretto I, Agostini M, Digito M (2012). Telomere-specific reverse transcriptase (hTERT) and cell-free RNA in plasma as predictors of pathologic tumor response in rectal cancer patients receiving neoadjuvant chemoradiotherapy. Ann Surg Oncol.

[38] Serilmez M, Ozgur E, Karaman S, Gezer U, Duranyildiz D (2019). Detection of serum protein and circulating mRNA of cMET, HGF EGF and EGFR levels in lung cancer patients to guide individualized therapy. Cancer Biomark A.

[39] Xue VW, Cheung MT, Chan PT, Luk LLY, Lee VH, Au TC (2019). Non-invasive potential circulating mRNA Markers for colorectal adenoma using targeted sequencing. Sci Rep.

[40] Tong Y, Zhao Z, Liu B, Bao A, Zheng H, Gu J (2018). 5’/ 3’ imbalance strategy to detect ALK fusion genes in circulating tumor RNA from patients with non-small cell lung cancer. J Exper Clin Cancer Res.

[41] Garcia V, Garcia JM, Silva J, Martin P, Pena C, Dominguez G (2009). Extracellular tumor-related mRNA in plasma of lymphoma patients and survival implications. PLoS ONE.

[42] Pasqualucci L, Dalla-Favera R (2018). Genetics of diffuse large B-cell lymphoma. Blood.

[43] Chambers J, Rabbitts TH (2015). LMO2 at 25 years: a paradigm of chromosomal translocation proteins. Open Biol.

[44] Zhao H, Kan Y, Wang X, Chen L, Ge P, Qian Z (2019). Genetic polymorphism and transcriptional regulation of CREBBP gene in patient with diffuse large B-cell lymphoma. Biosci Rep.

[45] Attia FM, Hassan AM, El-Maraghy NN, Ibrahium GH (2011). Clinical significance of suppressor of cytokines signalling-3 mRNA expression from patients with non-Hodgkin lymphoma under chemotherapy. Cancer Biomark A.

[46] Tamiya T, Kashiwagi I, Takahashi R, Yasukawa H, Yoshimura A (2011). Suppressors of cytokine signaling (SOCS) proteins and JAK/STAT pathways: regulation of T-cell inflammation by SOCS1 and SOCS3. Arterioscler Thromb Vasc Biol.

[47] Ujj Z, Buglyo G, Udvardy M, Vargha G, Biro S, Rejto L (2014). WT1 overexpression affecting clinical outcome in non-hodgkin lymphomas and adult acute lymphoblastic leukemia. Pathol Oncol Res.

[48] Yuan T, Huang X, Woodcock M, Du M, Dittmar R, Wang Y (2016). Plasma extracellular RNA profiles in healthy and cancer patients. Sci Rep.

[49] Jansson MD, Lund AH (2012). MicroRNA and cancer. Mol Oncol.

[50] Bartel DP (2009). MicroRNAs: target recognition and regulatory functions. Cell.

[51] Chen X, Ba Y, Ma L, Cai X, Yin Y, Wang K (2008). Characterization of microRNAs in serum: a novel class of biomarkers for diagnosis of cancer and other diseases. Cell Res.

[52] Kosaka N, Iguchi H, Ochiya T (2010). Circulating microRNA in body fluid: a new potential biomarker for cancer diagnosis and prognosis. Cancer Sci.

[53] Lawrie CH, Gal S, Dunlop HM, Pushkaran B, Liggins AP, Pulford K (2008). Detection of elevated levels of tumour-associated microRNAs in serum of patients with diffuse large B-cell lymphoma. Br J Haematol.

[54] Lopez-Santillan M, Larrabeiti-Etxebarria A, Arzuaga-Mendez J, Lopez-Lopez E, Garcia-Orad A (2018). Circulating miRNAs as biomarkers in diffuse large B-cell lymphoma: a systematic review. Oncotarget.

[55] Fang C, Zhu DX, Dong HJ, Zhou ZJ, Wang YH, Liu L (2012). Serum microRNAs are promising novel biomarkers for diffuse large B cell lymphoma. Ann Hematol.

[56] Borges NM, do Vale Elias M, Fook-Alves VL, Andrade TA, de Conti ML, Macedo MP (2016). Angiomirs expression profiling in diffuse large B-cell lymphoma. Oncotarget.

[57] Li J, Fu R, Yang L, Tu W (2015). miR-21 expression predicts prognosis in diffuse large B-cell lymphoma. Int J Clin Exp Pathol.

[58] Yuan WX, Gui YX, Na WN, Chao J, Yang X (2016). Circulating microRNA-125b and microRNA-130a expression profiles predict chemoresistance to R-CHOP in diffuse large B-cell lymphoma patients. Oncol Lett.

[59] Chen W, Wang H, Chen H, Liu S, Lu H, Kong D (2014). Clinical significance and detection of microRNA-21 in serum of patients with diffuse large B-cell lymphoma in Chinese population. Eur J Haematol.

[60] Zheng Z, Sun R, Zhao HJ, Fu D, Zhong HJ, Weng XQ (2019). MiR155 sensitized B-lymphoma cells to anti-PD-L1 antibody via PD-1/PD-L1-mediated lymphoma cell interaction with CD8 + T cells. Mol Cancer.

[61] Zheng Z, Xu PP, Wang L, Zhao HJ, Weng XQ, Zhong HJ (2017). MiR21 sensitized B-lymphoma cells to ABT-199 via ICOS/ICOSL-mediated interaction of Treg cells with endothelial cells. J Exper Clin Cancer Res.

[62] Meng Y, Quan L, Liu A (2018). Identification of key microRNAs associated with diffuse large B-cell lymphoma by analyzing serum microRNA expressions. Gene.

[63] Khare D, Goldschmidt N, Bardugo A, Gur-Wahnon D, Ben-Dov IZ, Avni B (2017). Plasma microRNA profiling: exploring better biomarkers for lymphoma surveillance. PLoS ONE.

[64] Inada K, Okoshi Y, Cho Y, Saito H, Iijima T, Hori M (2015). Availability of circulating MicroRNAs as a biomarker for early diagnosis of diffuse large B-cell lymphoma. Open J Blood Dis.

[65] Cui Q, Vari F, Cristino AS, Salomon C, Rice GE, Sabdia MB (2018). Circulating cell-free miR-494 and miR-21 are disease response biomarkers associated with interim-positron emission tomography response in patients with diffuse large B-cell lymphoma. Oncotarget.

[66] Jorgensen S, Paulsen IW, Hansen JW, Tholstrup D, Hother C, Sorensen E (2020). The value of circulating microRNAs for early diagnosis of B-cell lymphoma: A case-control study on historical samples. Sci Rep.

[67] Yan JW, Lin JS, He XX (2014). The emerging role of miR-375&nbsp;in cancer. Int J Cancer.

[68] Chou CH, Shrestha S, Yang CD, Chang NW, Lin YL, Liao KW (2018). miRTarBase update 2018: a resource for experimentally validated microRNA-target interactions. Nucleic Acids Res.

[69] Beheshti A, Stevenson K, Vanderburg C, Ravi D, McDonald JT, Christie AL (2019). Identification of circulating serum multi-microRNA signatures in human DLBCL models. Sci Rep.

[70] Tuncer SB, Akdeniz D, Celik B, Kilic S, Sukruoglu O, Avsar M (2019). The expression levels of miRNA-15a and miRNA-16-1&nbsp;in circulating tumor cells of patients with diffuse large B-cell lymphoma. Mol Biol Rep.

[71] Fabbri M, Bottoni A, Shimizu M, Spizzo R, Nicoloso MS, Rossi S (2011). Association of a microRNA/TP53 feedback circuitry with pathogenesis and outcome of B-cell chronic lymphocytic leukemia. JAMA.

[72] Aqeilan RI, Calin GA, Croce CM (2010). miR-15a and miR-16-1&nbsp;in cancer: discovery, function and future perspectives. Cell Death Differ.

[73] Thapa DR, Bhatia K, Bream JH, D’Souza G, Rinaldo CR, Wolinsky S (2012). B-cell activation induced microRNA-21 is elevated in circulating B cells preceding the diagnosis of AIDS-related non-Hodgkin lymphomas. AIDS.

[74] Barnes NA, Stephenson S, Cocco M, Tooze RM, Doody GM (2012). BLIMP-1 and STAT3 counterregulate microRNA-21 during plasma cell differentiation. J Immunol (Baltimore, Md: 1950).

[75] Medina PP, Nolde M, Slack FJ (2010). OncomiR addiction in an in vivo model of microRNA-21-induced pre-B-cell lymphoma. Nature.

[76] Iliopoulos D, Jaeger SA, Hirsch HA, Bulyk ML, Struhl K (2010). STAT3 activation of miR-21 and miR-181b-1 via PTEN and CYLD are part of the epigenetic switch linking inflammation to cancer. Mol Cell.

[77] Larrabeiti-Etxebarria A, Lopez-Santillan M, Santos-Zorrozua B, Lopez-Lopez E, Garcia-Orad A (2019). Systematic review of the potential of microRNAs in diffuse large B cell lymphoma. Cancers.

[78] Ahmadvand M, Eskandari M, Pashaiefar H, Yaghmaie M, Manoochehrabadi S, Khakpour G (2018). Over expression of circulating miR-155 predicts prognosis in diffuse large B-cell lymphoma. Leukemia Res.

[79] Bedewy AML, Elmaghraby SM, Shehata AA, Kandil NS (2017). Prognostic value of miRNA-155 expression in B-cell non-hodgkin lymphoma. Turk J Haematol.

[80] Bouvy C, Wannez A, George F, Graux C, Chatelain C, Dogne JM (2018). Circulating microRNAs as biomarkers in diffuse large b-cell lymphoma: a pilot prospective longitudinal clinical study. Biomark Cancer.

[81] Song G, Gu L, Li J, Tang Z, Liu H, Chen B (2014). Serum microRNA expression profiling predict response to R-CHOP treatment in diffuse large B cell lymphoma patients. Ann Hematol.

[82] Fajardo-Ramirez OR, Villela L, Campa-Carranza JN, Perez-Maya AA, Borrego-Soto G, Wah-Suarez MI (2020). miRNA signature associated with R-CHOP refractoriness in patients diagnosed with diffuse large B cell lymphoma. Non-coding RNA Res.

[83] Ting CY, Liew SM, Price A, Gan GG, Bee-Lan Ong D, Tan SY (2019). Clinical significance of aberrant microRNAs expression in predicting disease relapse/refractoriness to treatment in diffuse large B-cell lymphoma: A meta-analysis. Crit Rev Oncol Hematol.

[84] Sun R, Zheng Z, Wang L, Cheng S, Shi Q, Qu B (2020). A novel prognostic model based on 4 circulating miRNAs in diffuse large B-cell lymphoma: implications for the roles of MDSCs and Th17 cells in lymphoma progression. Mol Oncol.

[85] Wolf P (1967). The nature and significance of platelet products in human plasma. Br J Haematol.

[86] Gopal SK, Greening DW, Rai A, Chen M, Xu R, Shafiq A (2017). Extracellular vesicles: their role in cancer biology and epithelial-mesenchymal transition. Biochem J.

[87] Khawar MB, Abbasi MH, Siddique Z, Arif A, Sheikh N (2019). An update on novel therapeutic warfronts of extracellular vesicles (EVs) in cancer treatment: where we are standing right now and where to go in the future. Oxidative Med Cell Longev.

[88] Thery C, Witwer KW, Aikawa E, Alcaraz MJ, Anderson JD, Andriantsitohaina R (2018). Minimal information for studies of extracellular vesicles 2018 (MISEV2018): a position statement of the International Society for Extracellular Vesicles and update of the MISEV2014 guidelines. J Extracell Vesicles.

[89] Piper RC, Katzmann DJ (2007). Biogenesis and function of multivesicular bodies. Annu Rev Cell Dev Biol.

[90] Schorey JS, Bhatnagar S (2008). Exosome function: from tumor immunology to pathogen biology. Traffic.

[91] D’Souza-Schorey C, Clancy JW (2012). Tumor-derived microvesicles: shedding light on novel microenvironment modulators and prospective cancer biomarkers. Genes Dev.

[92] Rajagopal C, Harikumar KB (2018). The origin and functions of exosomes in cancer. Front Oncol.

[93] Domenyuk V, Zhong Z, Stark A, Xiao N, O’Neill HA, Wei X (2017). Plasma exosome profiling of cancer patients by a next generation systems biology approach. Sci Rep.

[94] Kalluri R, LeBleu VS (2020). The biology, function, and biomedical applications of exosomes. Science (New York, NY).

[95] Xiao XB, Gu Y, Sun DL, Ding LY, Yuan XG, Jiang HW (2019). Effect of rituximab combined with chemotherapy on the expression of serum exosome miR-451a in patients with diffuse large b-cell lymphoma. Eur Rev Med Pharmacol Sci.

[96] Di C, Jiang Y, Li M, Juan X, Xu C (2018). Circulating exosomal microRNA signature as a noninvasive biomarker for diagnosis of diffuse large B-cell lymphoma. Blood.

[97] Chang C, Liu J, He W, Qu M, Huang X, Deng Y (2018). A regulatory circuit HP1gamma/miR-451a/c-Myc promotes prostate cancer progression. Oncogene.

[98] Guo H, Nan Y, Zhen Y, Zhang Y, Guo L, Yu K (2016). miRNA-451&nbsp;inhibits glioma cell proliferation and invasion by downregulating glucose transporter 1. Tumour Biol.

[99] Rutherford SC, Fachel AA, Li S, Sawh S, Muley A, Ishii J (2018). Extracellular vesicles in DLBCL provide abundant clues to aberrant transcriptional programming and genomic alterations. Blood.

[100] Rutherford S, Fachel A, Sheng L, Yanwen J, Dominguez MP, Betel D (2017). DLBCL-derived exosomes provide key insights into genomic landscape in cell of origin and may lead to a novel method of surveillance and therapeutic intervention. Blood.

[101] Zare N, Haghjooy Javanmard S, Mehrzad V, Eskandari N, Kefayat A (2019). Evaluation of exosomal miR-155, let-7&nbsp;g and let-7&nbsp;i levels as a potential noninvasive biomarker among refractory/relapsed patients, responsive patients and patients receiving R-CHOP. Leukemia Lymphoma.

[102] Feng Y, Zhong M, Zeng S, Wang L, Liu P, Xiao X (2019). Exosome-derived miRNAs as predictive biomarkers for diffuse large B-cell lymphoma chemotherapy resistance. Epigenomics.

[103] Provencio M, Rodriguez M, Cantos B, Sabin P, Quero C, Garcia-Arroyo FR (2017). mRNA in exosomas as a liquid biopsy in non-Hodgkin Lymphoma: a multicentric study by the Spanish Lymphoma Oncology Group. Oncotarget.

[104] Koch R, Aung T, Vogel D, Chapuy B, Wenzel D, Becker S (2016). Nuclear trapping through inhibition of exosomal export by indomethacin increases cytostatic efficacy of doxorubicin and pixantrone. Clin Cancer Res.

[105] Aung T, Chapuy B, Vogel D, Wenzel D, Oppermann M, Lahmann M (2011). Exosomal evasion of humoral immunotherapy in aggressive B-cell lymphoma modulated by ATP-binding cassette transporter A3. Proc Natl Acad Sci USA.

[106] Ferguson Bennit HR, Gonda A, Oppegard LJ, Chi DP, Khan S, Wall NR (2017). Uptake of lymphoma-derived exosomes by peripheral blood leukocytes. Blood Lymphat Cancer Targets Ther.

[107] Chaput N, Thery C (2011). Exosomes: immune properties and potential clinical implementations. Semin Immunopathol.

[108] Zare N, Haghjooy Javanmard SH, Mehrzad V, Eskandari N, Andalib AR (2020). Effect of plasma-derived exosomes of refractory/relapsed or responsive patients with diffuse large B-cell lymphoma on natural killer cells functions. Cell J.

[109] Poggio M, Hu T, Pai CC, Chu B, Belair CD, Chang A (2019). Suppression of exosomal PD-L1 induces systemic anti-tumor immunity and memory. Cell.

[110] Chen Z, You L, Wang L, Huang X, Liu H, Wei JY (2018). Dual effect of DLBCL-derived EXOs in lymphoma to improve DC vaccine efficacy in vitro while favor tumorgenesis in vivo. J Exper Clin Cancer Res.

[111] Koch R, Demant M, Aung T, Diering N, Cicholas A, Chapuy B (2014). Populational equilibrium through exosome-mediated Wnt signaling in tumor progression of diffuse large B-cell lymphoma. Blood.

[112] Livshits MA, Khomyakova E, Evtushenko EG, Lazarev VN, Kulemin NA, Semina SE (2015). Isolation of exosomes by differential centrifugation: theoretical analysis of a commonly used protocol. Sci Rep.

[113] Van Deun J, Mestdagh P, Sormunen R, Cocquyt V, Vermaelen K, Vandesompele J, et al. The impact of disparate isolation methods for extracellular vesicles on downstream RNA profiling. J Extracell Vesicles. 2014;3. 10.3402/jev.v3.24858 PMC4169610 25317274

[114] Furi I, Momen-Heravi F, Szabo G (2017). Extracellular vesicle isolation: present and future. Ann Transl Med.

[115] Castillo J, Bernard V, San Lucas FA, Allenson K, Capello M, Kim DU (2018). Surfaceome profiling enables isolation of cancer-specific exosomal cargo in liquid biopsies from pancreatic cancer patients. Ann Oncol.

[116] Guttman M, Amit I, Garber M, French C, Lin MF, Feldser D (2009). Chromatin signature reveals over a thousand highly conserved large non-coding RNAs in mammals. Nature.

[117] Spizzo R, Almeida MI, Colombatti A, Calin GA (2012). Long non-coding RNAs and cancer: a new frontier of translational research?. Oncogene.

[118] Shi T, Gao G, Cao Y (2016). Long noncoding RNAs as novel biomarkers have a promising future in cancer diagnostics. Dis Mark.

[119] Mercer TR, Dinger ME, Mattick JS (2009). Long non-coding RNAs: insights into functions. Nat Rev Genet.

[120] Tayari MM, Winkle M, Kortman G, Sietzema J, de Jong D, Terpstra M (2016). Long noncoding RNA expression profiling in normal B-cell subsets and Hodgkin lymphoma reveals Hodgkin and Reed-Sternberg cell-specific long noncoding RNAs. Am J Pathol.

[121] Brazao TF, Johnson JS, Muller J, Heger A, Ponting CP, Tybulewicz VL (2016). Long noncoding RNAs in B-cell development and activation. Blood.

[122] Dahl M, Kristensen LS, Gronbaek K (2018). Long non-coding RNAs guide the fine-tuning of gene regulation in B-cell development and malignancy. Int J Mol Sci.

[123] Lu Z, Pannunzio NR, Greisman HA, Casero D, Parekh C, Lieber MR (2015). Convergent BCL6 and lncRNA promoters demarcate the major breakpoint region for BCL6 translocations. Blood.

[124] Verma A, Jiang Y, Du W, Fairchild L, Melnick A, Elemento O (2015). Transcriptome sequencing reveals thousands of novel long non-coding RNAs in B cell lymphoma. Genome Med.

[125] Sun J, Cheng L, Shi H, Zhang Z, Zhao H, Wang Z (2016). A potential panel of six-long non-coding RNA signature to improve survival prediction of diffuse large-B-cell lymphoma. Sci Rep.

[126] Zhou M, Zhao H, Xu W, Bao S, Cheng L, Sun J (2017). Discovery and validation of immune-associated long non-coding RNA biomarkers associated with clinically molecular subtype and prognosis in diffuse large B cell lymphoma. Mol Cancer.

[127] Gao HY, Wu B, Yan W, Gong ZM, Sun Q, Wang HH (2017). Microarray expression profiles of long non-coding RNAs in germinal center-like diffuse large B-cell lymphoma. Oncol Rep.

[128] Dousti F, Shahrisa A, Ansari H, Hajjari M, Tahmasebi Birgani Y, Mohammadiasl J (2018). Long non-coding RNAs expression levels in diffuse large B-cell lymphoma: an in silico analysis. Pathol Res Pract.

[129] Xu P, Chen X, Su P (2017). A pooled analysis of the clinical utilities of long non-coding RNA based molecular signature for diffuse large B cell lymphoma. Clin Lab.

[130] Qi P, Zhou XY, Du X (2016). Circulating long non-coding RNAs in cancer: current status and future perspectives. Mol Cancer.

[131] Isin M, Ozgur E, Cetin G, Erten N, Aktan M, Gezer U (2014). Investigation of circulating lncRNAs in B-cell neoplasms. Clin Chim Acta.

[132] Wang Y, Zhang M, Xu H, Wang Y, Li Z, Chang Y (2017). Discovery and validation of the tumor-suppressive function of long noncoding RNA PANDA in human diffuse large B-cell lymphoma through the inactivation of MAPK/ERK signaling pathway. Oncotarget.

[133] Peng W, Wu J, Feng J (2017). LincRNA-p21 predicts favorable clinical outcome and impairs tumorigenesis in diffuse large B cell lymphoma patients treated with R-CHOP chemotherapy. Clin Exper Med.

[134] Zhao S, Fang S, Liu Y, Li X, Liao S, Chen J (2017). The long non-coding RNA NONHSAG026900 predicts prognosis as a favorable biomarker in patients with diffuse large B-cell lymphoma. Oncotarget.

[135] Peng W, Feng J (2016). Long noncoding RNA LUNAR1 associates with cell proliferation and predicts a poor prognosis in diffuse large B-cell lymphoma. Biomed Pharmacother.

[136] Shi X, Cui Z, Liu X, Wu S, Wu Y, Fang F (2019). LncRNA FIRRE is activated by MYC and promotes the development of diffuse large B-cell lymphoma via Wnt/beta-catenin signaling pathway. Biochem Biophys Res Commun.

[137] Peng W, Fan H, Wu G, Wu J, Feng J (2016). Upregulation of long noncoding RNA PEG10 associates with poor prognosis in diffuse large B cell lymphoma with facilitating tumorigenicity. Clin Exper Med.

[138] Meng H, Zhao B, Wang Y (2020). FOXM1-induced upregulation of lncRNA OR3A4 promotes the progression of diffuse large B-cell lymphoma via Wnt/beta-catenin signaling pathway. Exp Mol Pathol.

[139] Peng W, Wu J, Feng J (2016). Long noncoding RNA HULC predicts poor clinical outcome and represents pro-oncogenic activity in diffuse large B-cell lymphoma. Biomed Pharmacother.

[140] Schmidt LH, Spieker T, Koschmieder S, Schaffers S, Humberg J, Jungen D (2011). The long noncoding MALAT-1 RNA indicates a poor prognosis in non-small cell lung cancer and induces migration and tumor growth. J thorac Oncol.

[141] Lai MC, Yang Z, Zhou L, Zhu QQ, Xie HY, Zhang F (2012). Long non-coding RNA MALAT-1 overexpression predicts tumor recurrence of hepatocellular carcinoma after liver transplantation. Med Oncol.

[142] Li LJ, Chai Y, Guo XJ, Chu SL, Zhang LS (2017). The effects of the long non-coding RNA MALAT-1 regulated autophagy-related signaling pathway on chemotherapy resistance in diffuse large B-cell lymphoma. Biomed Pharmacother.

[143] Gupta RA, Shah N, Wang KC, Kim J, Horlings HM, Wong DJ (2010). Long non-coding RNA HOTAIR reprograms chromatin state to promote cancer metastasis. Nature.

[144] Oh EJ, Kim SH, Yang WI, Ko YH, Yoon SO (2016). Long non-coding RNA HOTAIR expression in diffuse large B-cell lymphoma: in relation to polycomb repressive complex pathway proteins and H3K27 trimethylation. J Pathol Transl Med.

[145] Yan Y, Han J, Li Z, Yang H, Sui Y, Wang M (2016). Elevated RNA expression of long noncoding HOTAIR promotes cell proliferation and predicts a poor prognosis in patients with diffuse large B cell lymphoma. Mol Med Rep.

[146] Qian CS, Li LJ, Huang HW, Yang HF, Wu DP (2020). MYC-regulated lncRNA NEAT1 promotes B cell proliferation and lymphomagenesis via the miR-34b-5p-GLI1 pathway in diffuse large B-cell lymphoma. Cancer Cell Int.

[147] Deng L, Jiang L, Tseng KF, Liu Y, Zhang X, Dong R (2018). Aberrant NEAT1_1 expression may be a predictive marker of poor prognosis in diffuse large B cell lymphoma. Cancer Biomark A.

[148] Zhu Q, Li Y, Guo Y, Hu L, Xiao Z, Liu X (2019). Long non-coding RNA SNHG16 promotes proliferation and inhibits apoptosis of diffuse large B-cell lymphoma cells by targeting miR-497-5p/PIM1 axis. J Cell Mol Med.

[149] Cheng H, Yan Z, Wang X, Cao J, Chen W, Qi K (2019). Downregulation of long non-coding RNA TUG1 suppresses tumor growth by promoting ubiquitination of MET in diffuse large B-cell lymphoma. Mol Cell Biochem.

[150] Wang QM, Lian GY, Song Y, Huang YF, Gong Y (2019). LncRNA MALAT1 promotes tumorigenesis and immune escape of diffuse large B cell lymphoma by sponging miR-195. Life Sci.

[151] Zhao CC, Jiao Y, Zhang YY, Ning J, Zhang YR, Xu J (2019). Lnc SMAD5-AS1 as ceRNA inhibit proliferation of diffuse large B cell lymphoma via Wnt/beta-catenin pathway by sponging miR-135b-5p to elevate expression of APC. Cell Death Dis.

[152] Zhao L, Liu Y, Zhang J, Liu Y, Qi Q (2019). LncRNA SNHG14/miR-5590-3p/ZEB1 positive feedback loop promoted diffuse large B cell lymphoma progression and immune evasion through regulating PD-1/PD-L1 checkpoint. Cell Death Dis.

[153] Chen LY, Zhang XM, Han BQ, Dai HB (2020). Long noncoding RNA SNHG12 indicates the prognosis and accelerates tumorigenesis of diffuse large B-cell lymphoma through sponging microR-195. OncoTargets Ther.

[154] Huang X, Qian W, Ye X (2020). Long noncoding RNAs in diffuse large B-cell lymphoma: current advances and perspectives. OncoTargets Ther.

[155] Conn SJ, Pillman KA, Toubia J, Conn VM, Salmanidis M, Phillips CA (2015). The RNA binding protein quaking regulates formation of circRNAs. Cell.

[156] Memczak S, Jens M, Elefsinioti A, Torti F, Krueger J, Rybak A (2013). Circular RNAs are a large class of animal RNAs with regulatory potency. Nature.

[157] Pamudurti NR, Bartok O, Jens M, Ashwal-Fluss R, Stottmeister C, Ruhe L (2017). Translation of CircRNAs. Mol Cell.

[158] Jeck WR, Sorrentino JA, Wang K, Slevin MK, Burd CE, Liu J (2013). Circular RNAs are abundant, conserved, and associated with ALU repeats. RNA.

[159] Kosik KS (2013). Molecular biology: circles reshape the RNA world. Nature.

[160] Nicolet BP, Engels S, Aglialoro F, van den Akker E, von Lindern M, Wolkers MC (2018). Circular RNA expression in human hematopoietic cells is widespread and cell-type specific. Nucleic Acids Res.

[161] Kristensen LS, Hansen TB, Veno MT, Kjems J (2018). Circular RNAs in cancer: opportunities and challenges in the field. Oncogene.

[162] Dahl M, Daugaard I, Andersen MS, Hansen TB, Gronbaek K, Kjems J (2018). Enzyme-free digital counting of endogenous circular RNA molecules in B-cell malignancies. Lab Invest.

[163] Chen X, Xie X, Zhou W (2020). CircCFL1/MiR-107 axis targeting HMGB1 promotes the malignant progression of diffuse large B-cell lymphoma tumors. Cancer Manag Res.

[164] Dzikiewicz-Krawczyk A, Kok K, Slezak-Prochazka I, Robertus JL, Bruining J, Tayari MM (2017). ZDHHC11 and ZDHHC11B are critical novel components of the oncogenic MYC-miR-150-MYB network in Burkitt lymphoma. Leukemia.

[165] Yang Q, Du WW, Wu N, Yang W, Awan FM, Fang L (2017). A circular RNA promotes tumorigenesis by inducing c-myc nuclear translocation. Cell Death Differ.

[166] Guarnerio J, Bezzi M, Jeong JC, Paffenholz SV, Berry K, Naldini MM (2016). Oncogenic role of fusion-circRNAs derived from cancer-associated chromosomal translocations. Cell.

[167] Hu Y, Zhao Y, Shi C, Ren P, Wei B, Guo Y (2019). A circular RNA from APC inhibits the proliferation of diffuse large B-cell lymphoma by inactivating Wnt/β-catenin signaling via interacting with TET1 and miR-888. Aging.

[168] Chen DF, Zhang LJ, Tan K, Jing Q (2018). Application of droplet digital PCR in quantitative detection of the cell-free circulating circRNAs. Biotechnol Biotechnol Equip.

[169] Leslie M (2010). Cell biology. Beyond clotting: the powers of platelets. Science.

[170] Sol N, In 't Veld S, Vancura A, Tjerkstra M, Leurs C, Rustenburg F (2020). Tumor-educated platelet RNA for the detection and (pseudo)progression monitoring of glioblastoma. Cell Rep Med.

[171] Marchesi F, Regazzo G, Palombi F, Terrenato I, Sacconi A, Spagnuolo M (2018). Serum miR-22 as potential non-invasive predictor of poor clinical outcome in newly diagnosed, uniformly treated patients with diffuse large B-cell lymphoma: an explorative pilot study. J Exper Clin Cancer Res.

[172] Caivano A, La Rocca F, Simeon V, Girasole M, Dinarelli S, Laurenzana I (2017). MicroRNA-155 in serum-derived extracellular vesicles as a potential biomarker for hematologic malignancies—a short report. Cell Oncol.

[173] Zare N, Eskandari N, Mehrzad V, Javanmard SH (2019). The expression level of hsa-miR-146a-5p in plasma-derived exosomes of patients with diffuse large B-cell lymphoma. J Res Med Sci.

